# Transformers for fMRI analysis: from spatiotemporal modeling to brain foundation models

**DOI:** 10.3389/fninf.2026.1869687

**Published:** 2026-10-07

**Authors:** Xiaomin Yang, Yue Li, Xue Zhang, Bocheng Wang

**Affiliations:** School of Media Engineering, Communication University of Zhejiang, Hangzhou, Zhejiang, China

**Keywords:** brain functional representation, fMRI, self-supervised learning, spatio-temporal modeling, Transformer

## Abstract

Functional magnetic resonance imaging (fMRI) serves as a pivotal tool for investigating the functional organization and dynamic connectivity of the human brain, indirectly reflecting neural activity through blood oxygen level-dependent (BOLD) signals. However, the high-dimensional spatiotemporal structure, prominent noise interference, and inter-individual variability of fMRI data pose significant challenges for traditional modeling methods in characterizing complex brain functional patterns. In recent years, the Transformer architecture has gained significant traction in fMRI data analysis, leveraging its self-attention mechanism to model global dependencies. It has demonstrated superior performance in tasks such as brain disease classification, functional connectivity modeling, and brain representation learning. Compared to convolutional and recurrent neural networks, Transformers can more effectively capture long-range spatiotemporal dependencies and offer stronger structural scalability and cross-task transfer potential. To systematically map the development of this field, this paper proposes a unified taxonomy for Transformer-fMRI methods based on modeling dimensions. It categorizes existing research into four primary paradigms: temporal modeling, spatial modeling, spatiotemporal joint modeling, and self-supervised learning, providing a comparative analysis of their structural characteristics and applicable scenarios. Furthermore, this paper summarizes the key challenges regarding computational complexity, data dependency, and interpretability, while exploring the integration of neuroscientific priors with large-scale pre-trained models. Finally, we discuss future directions for Transformers in the construction of brain foundation models and their clinical utility.

## 1 Introduction

### 1.1 Research background on fMRI data analysis

Functional magnetic resonance imaging (fMRI) is a non-invasive brain imaging technique that indirectly reflects neural activity through blood oxygen level-dependent (BOLD) signals (Kwong et al., 1992). It has been widely used to investigate complex cognitive functions and their dynamic organizational patterns in the human brain (Menon, 2023; Gordon et al., 2023; Zhao, 2025). However, modeling fMRI data remains fundamentally challenging due to their high-dimensional and dynamically coupled spatiotemporal structure (Iraji et al., 2019). These properties pose significant challenges for traditional methods in representing complex patterns of brain function, resulting in a mismatch between the intrinsic complexity of fMRI data and the capabilities of conventional modeling approaches (Abrol et al., 2021; Wang et al., 2023).

As a representative four-dimensional dataset comprising three spatial dimensions and one temporal dimension, fMRI captures spatially organized functional networks constrained by anatomical structures while also characterizing dynamic interactions among brain regions that evolve with changing cognitive states over time (Pang et al., 2023; Deco et al., 2021). This suggests that the brain is neither a static “wiring diagram” nor merely a collection of independent time series, but rather a complex dynamic system characterized by strong spatial coupling and continuous temporal evolution (Lynn and Bassett, 2019). Consequently, effective models should simultaneously capture spatial cooperative relationships and their temporal dynamics.

However, conventional methods often focus on only a single dimension. Generalized linear models (GLMs) and static functional connectivity (sFC) typically assume linear responses or aggregate temporal data over the entire scan, thereby overlooking dynamic characteristics (Wu et al., 2024; Preti et al., 2017). While FC can reveal statistical dependencies, it struggles to distinguish between direct causal interactions and spurious co-activations and is further limited by static assumptions, making it incapable of capturing complex network reorganization associated with changing cognitive states.

To enhance nonlinear modeling capabilities, deep learning has been widely adopted (Chen et al., 2022; Avberšek and Repovš, 2022; Munroe et al., 2024), but it still faces several challenges: spatially, the local receptive fields of CNNs struggle to model long-range global interactions in brain networks (Han et al., 2023; Bessadok et al., 2023; Shamshad et al., 2023; Seguin et al., 2023; Faskowitz et al., 2022; Tang et al., 2026), while GNNs are constrained by predefined graph structures and issues of over-smoothing (Cui et al., 2023); temporally, RNNs face challenges in modeling long sequences (Bai et al., 2018), and most methods adopt separate spatial and temporal modeling, overlooking the intrinsic coupling between the two. Furthermore, the “few-shot, high-dimensional” nature of the data and cross-domain shifts across centers further constrain model generalization (Marek et al., 2022; Sadil and Lindquist, 2026; Lee et al., 2024). Consequently, developing an analytical framework capable of jointly modeling complex spatiotemporal dependencies while maintaining strong generalization has become a key research direction (Gadgil et al., 2020).

### 1.2 Applications and advantages of Transformers in fMRI

The Transformer architecture was originally proposed by Vaswani et al. (2017) in the field of natural language processing, using self-attention mechanisms to model global dependencies in sequences. Subsequently, the success of the Vision Transformer (ViT) (Dosovitskiy et al., 2021) further validated its versatility in modeling high-dimensional spatial data and drove its widespread adoption in the field of medical imaging (Chen et al., 2021; He et al., 2023).

In the field of fMRI, researchers have begun to incorporate Transformers into time-series and brain network modeling, yielding promising early results. For example, the BolT model (Bedel et al., 2023) employs a windowed attention mechanism to model BOLD time series from local to global scales; the STAGIN architecture (Kim et al., 2021) introduces spatio-temporal self-attention into dynamic functional connectivity learning, effectively capturing the evolution of brain states. These studies have demonstrated the effectiveness of attention mechanisms in modeling neural activity patterns and have contributed to the development of foundation models for brain imaging (Dong Z. et al., 2025; Wei et al., 2025).

Overall, the advantages of Transformers in fMRI applications are primarily reflected in three aspects. First, the self-attention mechanism can model long-range dependencies across the entire brain; unlike the local receptive fields of traditional CNNs, it is better suited for modeling functional coordination between distant brain regions (Zhou et al., 2024; Yu, 2026), and can implicitly learn dynamic functional connectivity structures (Kan et al., 2022a; Li et al., 2021). Second, Transformers can jointly model spatial, temporal, and individual-level information (Kazemivash et al., 2025; Ren et al., 2024; Wang et al., 2024), overcoming the fragmented processing of different dimensions seen in traditional methods (Thapaliya et al., 2025; Shehzad et al., 2025; Zhang et al., 2024). Finally, Transformers are highly compatible with self-supervised learning, enabling the learning of general brain representations when labeled data are scarce. Large-scale pre-trained models (such as BrainGFM) have demonstrated strong generalization capabilities across various brain disease tasks (Leng et al., 2025; Shi et al., 2023; Wei et al., 2026; Malkiel et al., 2022).

It should be noted that Transformers are not optimal in all fMRI scenarios. Their self-attention mechanisms have an *O*(*N*^2^) computational complexity, resulting in high computational overhead (Farina et al., 2024), and they lack explicit inductive biases, which may lead to overfitting risks in small-sample settings (Lin et al., 2022). Therefore, their effective application still depends on robust tokenization strategies and efficient attention designs.

### 1.3 Scope and contributions of the study

In recent years, the Transformer architecture has been widely applied in tasks such as fMRI-based brain disease classification (Song et al., 2025), brain functional representation learning (Zhou et al., 2024), and cross-subject generalization (Zongxin and Lin, 2024). However, existing studies have primarily focused on improving performance in downstream tasks (Bhavna et al., 2024), lacking a unified analytical perspective on the deep spatiotemporal structure of fMRI and the representation of individual differences (Gao et al., 2025). To address this, this paper conducts a structured review of existing research from the perspective of “modeling dimensions,” focusing on how different designs address the inherent high-dimensionality, spatiotemporal coupling, and dynamic complexity of fMRI.

Specifically, current models differ significantly in their assumptions about the underlying data structure: one line of research treats fMRI as a collection of brain-region time series to model long-range temporal dependencies (Zhou et al., 2025; Yu et al., 2026); another draws inspiration from Vision Transformers, partitioning the data into patches or tokens to perform global modeling of spatial or spatiotemporal information (Jung et al., 2025; Kannan et al., 2025). Although each approach has its own advantages, their underlying assumptions, scope of applicability, and potential risks of information loss necessitate systematic comparative analysis (Wang et al., 2026b).

The core contributions of this paper are as follows: First, we categorize and compare modeling frameworks based on temporal, spatial, and spatiotemporal joint modeling, analyzing their differences in computational cost and information retention. Second, we review self-supervised paradigms such as masked modeling and contrastive learning, as well as their critical roles in constructing foundation models of brain function. Third, we summarize the challenges these models face in terms of computational cost and interpretability, and identify future directions for integrating neuroscientific prior knowledge.

To clearly illustrate the aforementioned technological trajectory, this paper constructs a comprehensive logical framework for Transformer-based fMRI analysis (as shown in Figure 1). This figure integrates existing fragmented research and clearly delineates three major stages: data representation (preprocessing and tokenization), the evolution of core modeling paradigms, and clinical translation. It serves as a guiding framework throughout the paper.

**Figure 1:**
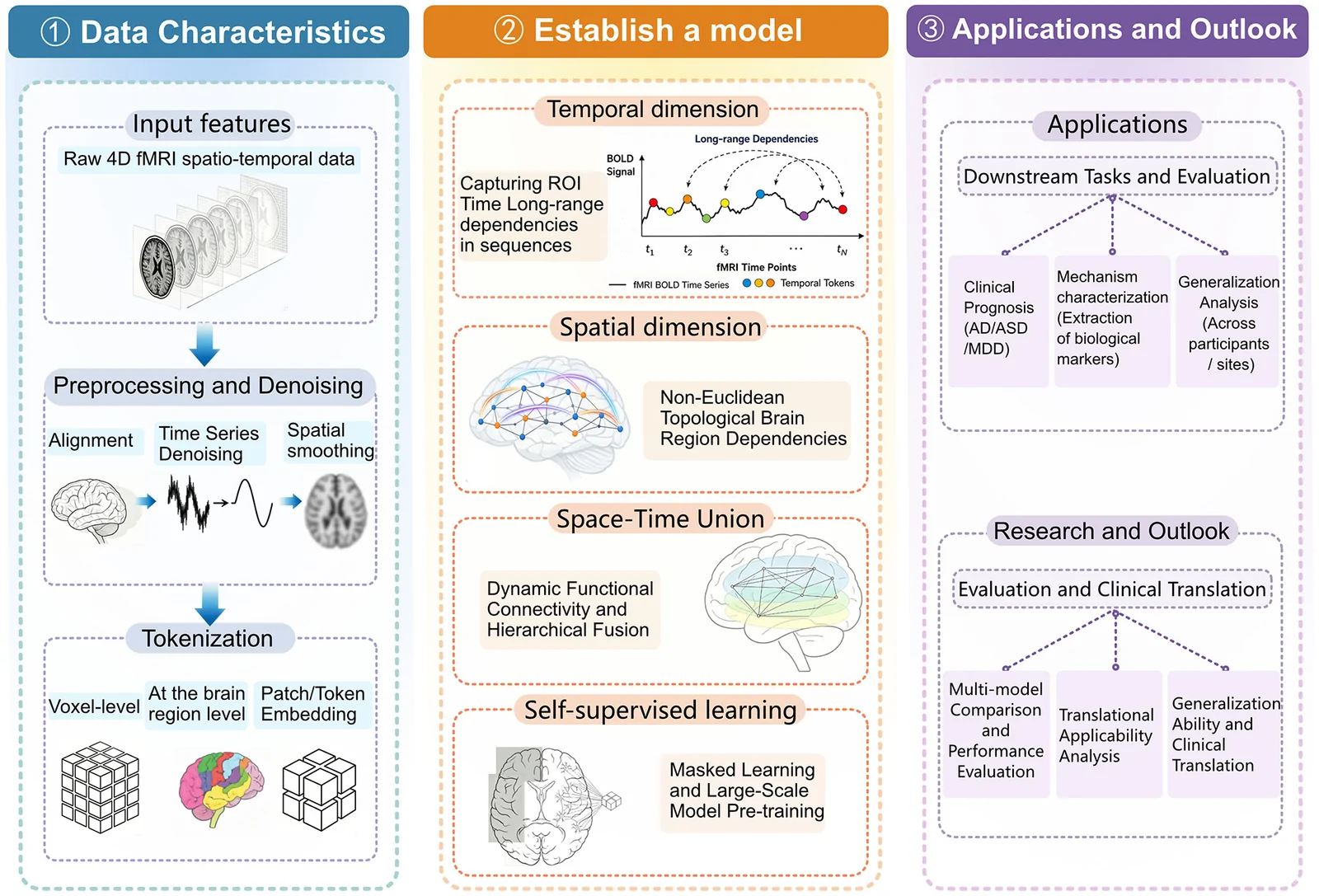
An overview of the Transformer-based fMRI analysis framework. This figure systematically outlines the end-to-end workflow of Transformers in fMRI analysis, covering three core stages: (1) data representation (preprocessing and tokenization), (2) modeling paradigms (temporal, spatial, spatiotemporal, and self-supervised modeling), and (3) applications and outlook (clinical translation and future challenges).

## 2 FMRI data preprocessing and data representation

### 2.1 Characteristics of fMRI data and modeling challenges

Functional magnetic resonance imaging (fMRI) data are typically represented as a four-dimensional tensor comprising three spatial dimensions and one temporal dimension. In this structure, each voxel corresponds to a time-varying BOLD signal sequence, collectively constituting high-dimensional spatiotemporal data. However, from a modeling perspective, the raw data present several challenges for effective modeling.

First, fMRI data are characterized by high dimensionality and a low signal-to-noise ratio; a single scan typically contains tens of thousands to hundreds of thousands of voxels, but the proportion of neural signals of interest is relatively low, making the data susceptible to factors such as head motion, physiological noise, and scanner drift (Lindquist et al., 2019). Second, fMRI time-series signals exhibit complex dependencies, featuring not only short-term local correlations but also long-term dynamic evolution patterns across scales (Ding et al., 2026). Furthermore, individual differences in brain function among participants and the heterogeneity of multi-center scanning protocols can induce significant distribution shifts, further limiting the model's cross-domain generalization performance (Dadi et al., 2019).

Due to the factors mentioned above, raw voxel-level fMRI data are difficult to use directly in deep learning modeling. On the one hand, the high-dimensional nature of the data imposes a significant computational burden; on the other hand, noise may interfere with the model's ability to learn meaningful neural patterns. Therefore, before proceeding to downstream tasks, the data must undergo structural reconstruction and information compression through standardized preprocessing and appropriate representation methods.

In summary, the high dimensionality, complex noise, and potential distribution shifts across individuals and scanning sites in fMRI data collectively necessitate standardized preprocessing and robust data representation, which form the foundation for building effective fMRI-Transformer models.

### 2.2 Standardized preprocessing and signal denoising

To reduce noise and scanning-related variability in raw fMRI data, standardized preprocessing pipelines have become a fundamental consensus in neuroimaging analysis. Modern research typically divides this process into two complementary modules: structural image (T1-weighted) preprocessing and functional image (BOLD) preprocessing.

During the structural image preprocessing stage, the primary objective is to construct a high-quality anatomical reference space, which involves skull stripping, tissue segmentation, and nonlinear spatial normalization to a standard brain template (such as MNI space). These steps provide a unified spatial reference frame for subsequent cross-subject analyses and form the foundation for population-level statistics and machine learning modeling. In recent years, automated and deep learning-driven tools (such as DeepPrep) have further enhanced the efficiency and robustness of structural image preprocessing, enabling the processing of large-scale datasets (Ren et al., 2025).

Functional image preprocessing primarily focuses on temporal consistency and the correction of motion artifacts. A typical workflow includes steps such as slice timing correction, motion correction, and functional-to-structural co-registration to reduce sampling errors and systematic biases introduced by subject motion. In addition, spatial normalization maps functional images to a unified standard space, thereby enhancing the comparability of data across individuals. Previous studies have shown that different preprocessing pipelines can significantly impact final performance in connectome modeling and classification tasks, though the overall trend still demonstrates strong robustness (Medvedeva et al., 2026).

During the signal denoising stage, methods such as regressing out white matter and cerebrospinal fluid (CSF) signals, bandpass filtering, and spatial smoothing are commonly employed to reduce noise interference from non-neural sources. While these processing steps improve the signal-to-noise ratio, they may also affect the representation of fine-grained functional connectivity information; therefore, a trade-off between signal cleaning and information preservation must be considered in machine learning tasks. Studies based on standardized pipelines (such as fMRIPrep) and their extended versions have shown that a unified preprocessing strategy helps improve the stability and generalization ability of models across datasets (Ren et al., 2025).

In summary, standardized preprocessing workflows systematically reconstruct the spatiotemporal structure of fMRI signals, providing a reliable data foundation for subsequent representation learning and deep learning model training, and have become a critical preliminary step in current machine learning research in neuroimaging.

### 2.3 Tokenization strategies for fMRI data

Preprocessed fMRI data must be converted into a structured sequence representation to meet the input requirements of the Transformer. Based on spatial granularity, mainstream methods can be categorized into three types: voxel-level, region-of-interest (ROI)-level, and functional connectivity (FC)-based.

Voxel-level representations directly use voxel signals or local patches in three-dimensional space as input units, thereby preserving local details to the greatest extent possible and avoiding the structural bias introduced by predefined atlases. However, their high-dimensional nature results in significant computational and memory overhead. To address this, recent studies have adopted patch-based embedding or atlas-free strategies to reduce the number of tokens and improve modeling feasibility. For example, Omni-fMRI explores a unified representation learning paradigm under atlas-free conditions (Wang et al., 2026c).

Region-of-interest (ROI) representations are the most widely used paradigm in current Transformer-based brain modeling. Based on classic brain atlases including AAL, Schaefer, and Glasser, this method aggregates voxel signals into time series of multiple ROIs, where each ROI corresponds to a single token. Such a design substantially reduces the sequence length and strengthens the capability to model whole-brain functional dependencies. For brain disease prediction tasks (e.g., autism), multi-scale ROI Transformers have been proven to effectively extract local features within functional brain networks and fuse global information across whole-brain networks, thus greatly boosting classification performance (Bannadabhavi et al., 2023).

Functional connectivity (FC) representations involve generating a connectivity matrix that reflects interactions between brain regions by calculating statistical dependencies (such as Pearson correlations) among ROI time series. This type of representation focuses on network topology rather than the raw time series themselves.

The three methods described above essentially involve mapping continuous spatiotemporal signals to discrete token sequences, and the choice of method directly impacts the Transformer's ability and efficiency in modeling brain functional representations. A well-designed data representation is not merely the endpoint of preprocessing, but also the core foundation for token design and downstream performance. Figure 2 illustrates the complete transformation path from raw 4D fMRI data to Transformer input tokens, encompassing structural image normalization, functional image correction, signal denoising, and three types of feature construction (voxel patches, ROI sequences, and FC matrices). It visually demonstrates the differences in spatial granularity and topological structure among various representations, providing structured inputs for subsequent modeling.

**Figure 2:**
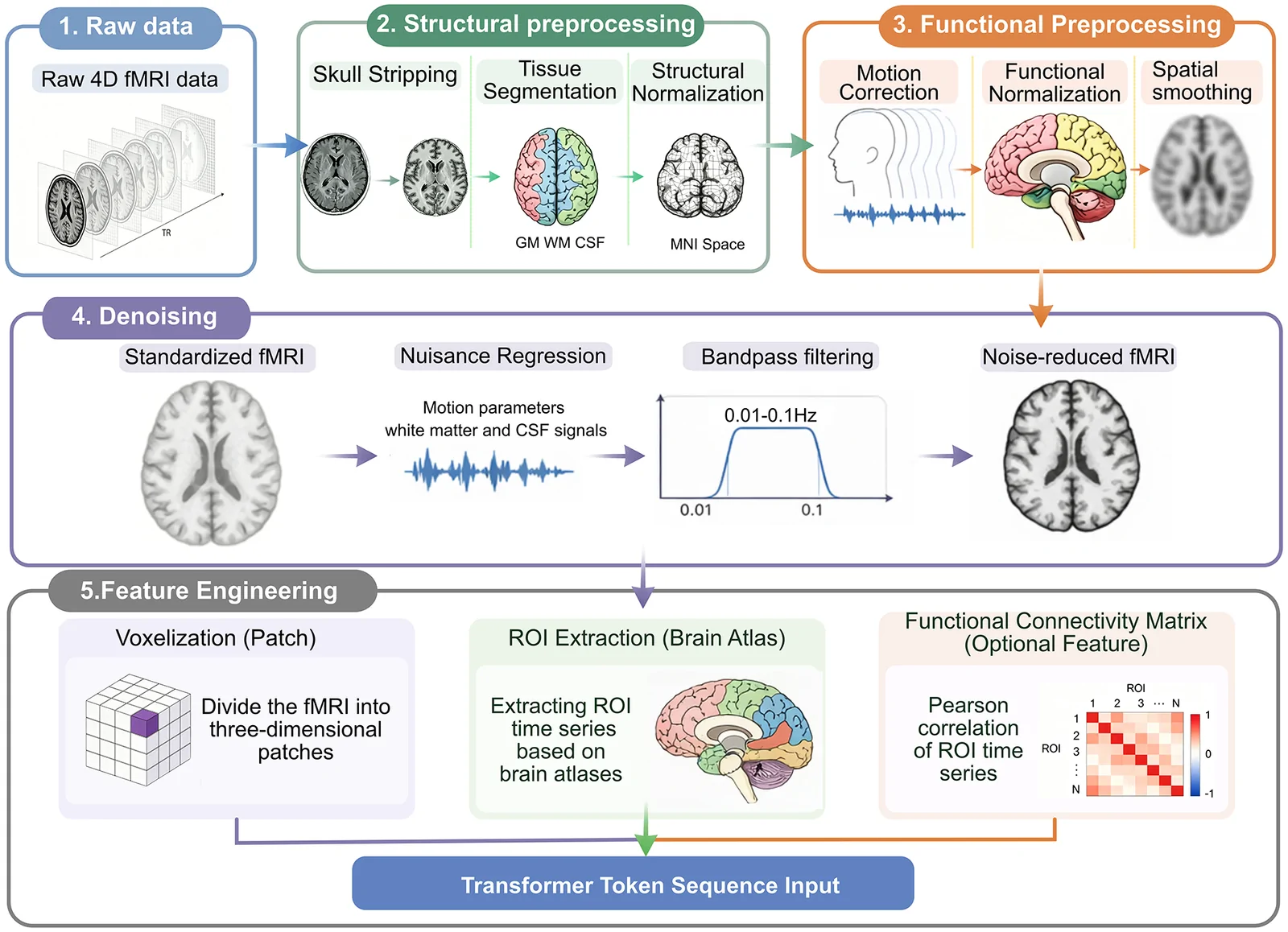
Processing workflow from raw 4D fMRI data to Transformer input tokens: (1) Raw 4D fMRI data; (2) Structural image preprocessing and normalization; (3) Head motion correction and smoothing of functional images; (4) Removal of physiological noise and signal drift through nuisance regression and temporal filtering; (5) Feature engineering to construct three types of features, voxel patches, ROI time series, and functional connectivity matrices, and conversion into token sequences.

## 3 Transformer modeling paradigms for fMRI

The Transformer architecture has been applied in various ways to fMRI analysis, with the key distinction lying in how high-dimensional brain imaging data are mapped onto a sequence of tokens. Based on the feature dimensions captured by the model, we classify existing modeling paradigms into three categories: temporal, spatial, and joint spatiotemporal modeling.

### 3.1 Temporal transformer modeling

Temporal Transformer modeling approaches primarily treat fMRI data as multivariate time-series signals. These methods typically ignore or downplay explicit spatial structures, focusing instead on the dynamic changes in brain signals over time. This modeling approach aligns with traditional time-series analysis methods but significantly enhances the ability to model long-range dependencies through self-attention mechanisms.

#### 3.1.1 ROI temporal token construction

In the temporal modeling paradigm, researchers typically begin by parcellating fMRI data into brain regions. They use predefined brain atlases [such as AAL, Schaefer (Schaefer et al., 2018), or the Yeo networks (Thomas Yeo et al., 2011)] to convert four-dimensional fMRI data into an ROI-level time-series matrix. Under this representation, each brain region corresponds to a time series, thereby simplifying the raw data into an *R*×*T* matrix (where *R* is the number of brain regions and *T* is the number of time points).

In Transformer models, researchers typically treat the whole-brain ROI activation vector at a single time point as a single token. Each token represents the brain's overall functional state at a given time point. These tokens are then fed into the Transformer encoder in temporal order, thereby learning dependencies across the temporal dimension (Khajehnejad et al., 2025).

This approach to token construction has a clear physical correspondence: each token aligns with a real fMRI temporal sample acquired at one repetition time (TR). This design enables the model to directly capture the temporal evolution of the BOLD signal without the need for complex spatial embedding strategies.

#### 3.1.2 Long-range attention advantage

In time-based Transformers, the core feature extraction engine is the Multi-Head Self-Attention (MHSA) mechanism. As mentioned earlier, the key to the Transformer's ability to overcome the limitations of fixed topological priors lies in the implicit graph inference capabilities of its attention matrix. Specifically, in pure temporal modeling scenarios, when sequence tokens representing brain-region states at each time point are input into the model, the self-attention mechanism is not restricted to local dependencies between adjacent time points. Instead, it generates a *T*×*T* temporal attention weight matrix by computing the dot product of query and key vectors (Vaswani et al., 2017). From a functional perspective, this implicitly constructed attention matrix can be interpreted as a representation of temporal dependencies between different time points within a scanning cycle (Mahmood et al., 2023; Zhang et al., 2025b).

Thanks to its global receptive field, the Transformer can directly model dependencies between temporally distant time points without relying on a predefined fixed-length temporal window (de Alteriis et al., 2025). This is particularly relevant for characterizing biologically meaningful slow-frequency fluctuations (typically below 0.1 Hz) in fMRI data. Through weighted aggregation of information across the sequence, the model can flexibly represent these slow-frequency dynamics, which may include transient synchronization between different functional networks (Esteban et al., 2026) and spontaneous microstate transitions during rest (Islam et al., 2024).

Furthermore, the multi-head mechanism allows the model to simultaneously uncover temporal patterns across different representational subspaces. This design naturally aligns with the multiscale dynamic characteristics of the brain when processing complex cognitive processes: some attention heads may focus on capturing short-term, high-frequency local transient responses, while others may be dedicated to extracting global state drift patterns spanning the entire scanning cycle. Consequently, compared to traditional sliding-window methods, self-attention-based temporal feature extraction not only avoids the dilemma of selecting an appropriate window length but also enables adaptive modeling of the brain's long-term dynamic dependencies in a fully data-driven manner.

#### 3.1.3 Typical temporal transformers and task adaptation

In recent years, temporal Transformers have demonstrated strong performance in fMRI tasks such as decoding brain states, classifying mental disorders, and self-supervised feature learning.

In brain state decoding, pure temporal modeling can directly capture non-stationary features that evolve over time. For example, Sun et al. (2025a) utilized causal attention masks to prevent information leakage and achieved autoregressive prediction of future BOLD signals; Kwon et al. (2025) successfully predicted task-state activation patterns by extracting resting-state temporal dependencies.

In the task of classifying psychiatric and neurological disorders, controlling model complexity to prevent overfitting is crucial due to the small sample size and high heterogeneity of clinical data. To improve the efficiency of long-sequence modeling, the BolT architecture proposed by Bedel et al. (2023) (as shown in Figure 3) introduces Fusion Window Multi-Head Attention (FW-MSA), achieving accurate classification while reducing computational complexity. Following this approach, Dai et al. (2024) combined local time windows with attention pruning to extract discriminative features, achieving efficient classification of major depressive disorder (MDD). Meanwhile, cross-modal weight transfer has also shown considerable potential: Wang (2025) parcellated the fMRI data into 360 ROIs using a multimodal cortical atlas and, in their optimal TS-wise strategy, constructed a Patch Token from the features of all ROIs at a single time point, thereby adapting ViT pre-trained weights to fMRI time-series data. Similarly, Sarraf et al. (2023) significantly reduced the number of parameters by 30% through custom linear projections in their OViTAD model, with both approaches achieving significant improvements in the classification of mild cognitive impairment (MCI) and Alzheimer's disease (AD).

**Figure 3:**
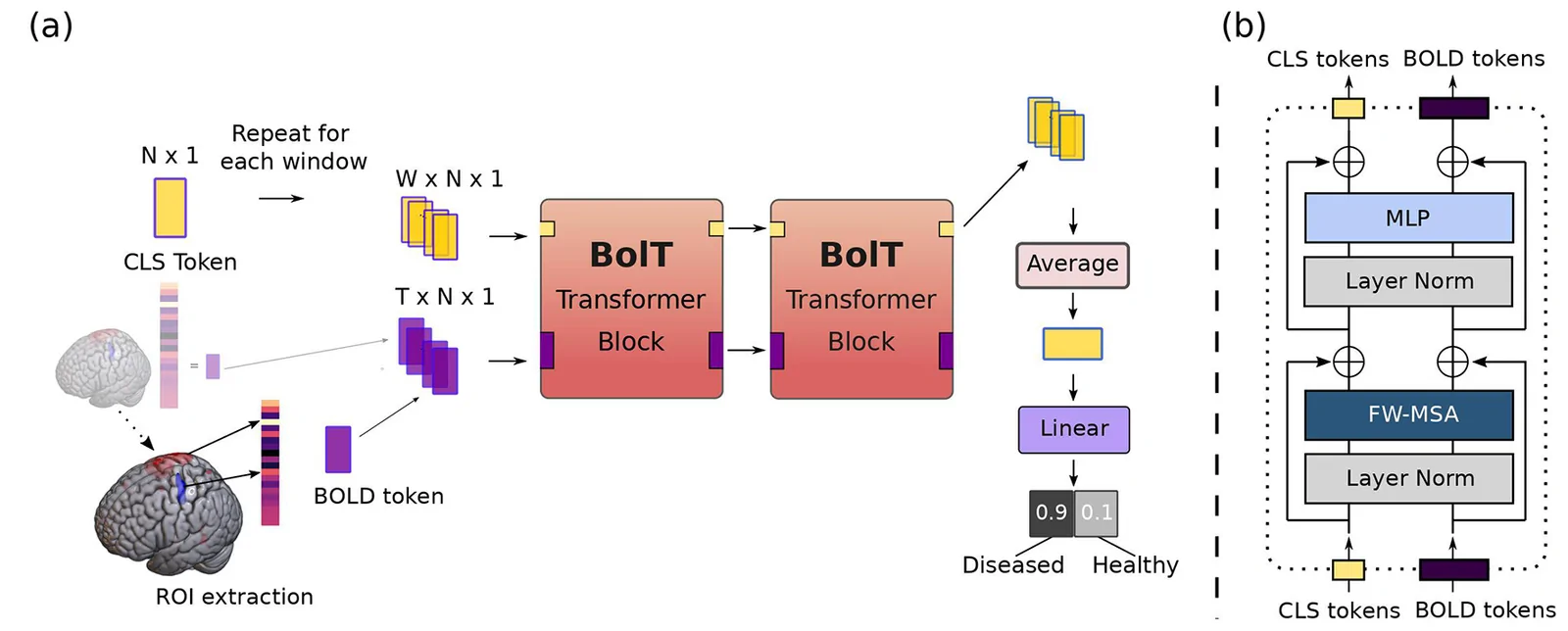
Adaptation of a typical time-domain fMRI Transformer architecture to classification tasks (BolT). **(a)** The overall BolT classification pipeline; **(b)** Internal structure of the BolT Transformer block. The model converts ROI time series into BOLD tokens, extracts temporal features via cascaded windowed attention, and ultimately uses the CLS token to determine the state. Figure reused from the BolT GitHub repository (https://github.com/icon-lab/BolT) with written permission from the original authors.

In the fields of self-supervised learning and foundational models, researchers are leveraging vast amounts of unlabeled data to explore the underlying temporal dynamics of the brain. Ferrante et al. (2025) introduced masked autoencoders (MAEs) to reconstruct time series and capture neural dynamics; Han et al. (2025) constructed a hypergraph foundation model that demonstrated excellent few-shot transfer capabilities in downstream disease diagnosis. To balance computational feasibility, the BrainNPT framework (Hu et al., 2024) employs ROI-based masking prediction, preserving global connectivity while avoiding the massive memory overhead associated with voxel-level modeling.

Overall, these representative studies demonstrate the expressive capabilities and task adaptability of temporal Transformers, while also highlighting the importance of architectural design for different fMRI analysis tasks.

#### 3.1.4 Pure temporal modeling and spatial representation bottleneck

The primary advantages of temporal Transformers lie in their simple structure and manageable computational cost; their design avoids complex spatial modeling mechanisms, making them well-suited for fMRI research scenarios with limited sample sizes (Li and Wang, 2026). Due to their relatively straightforward architecture and stable training process, this paradigm is particularly well-suited for downstream tasks centered on temporal dynamics, such as brain state decoding, disease classification, or cross-temporal state prediction (Jeyabose et al., 2025). However, its limitations are equally significant: such methods typically compress whole-brain spatial information into ROI activation vectors at each time point, failing to explicitly model anatomical proximity, functional hierarchies, or known network topologies between brain regions. Consequently, they have limited capabilities in interpreting specific functional connectivity patterns or identifying interactions among brain networks. Furthermore, although attention weights are often used to infer dependencies between brain regions, existing research has explicitly pointed out that attention mechanisms essentially reflect computational allocation strategies within the model rather than true neural causality or physiological mechanisms (Jain and Wallace, 2019); therefore, relying solely on attention weights for neuroscientific interpretation carries a risk of misinterpretation and requires validation through other interpretability methods or domain-specific prior knowledge.

Overall, temporal Transformer modeling methods provide an effective paradigm for fMRI time-series analysis. These methods demonstrate clear advantages in capturing long-range temporal dependencies and dynamic brain states, and have achieved promising results in tasks such as brain state prediction, mental disorder classification, and self-supervised representation learning. However, their ability to capture spatial structure is limited. This limitation has directly driven the development of subsequent spatial modeling and spatiotemporal joint modeling methods.

### 3.2 Spatial Transformer modeling

Unlike the temporal dimension, which focuses on capturing the dynamic evolution of brain activity, the spatial modeling paradigm treats fMRI data as a three-dimensional spatial structure or topological network. The core objective of this paradigm is to address the question of “how different brain regions work together”–that is, to reconstruct the brain's functional connectivity architecture by capturing the dependencies between different spatial locations (brain regions or voxels).

#### 3.2.1 Spatial token construction from ROIs to 3D patches

Under this paradigm, the primary challenge is how to map the three-dimensional brain space onto sequences that a Transformer can process. Current solutions fall into two main categories:

Region-of-interest (ROI)-based: This approach best aligns with neuroscientific intuition. It uses a priori brain atlases to partition the brain into *N* regions of interest (ROIs), with each ROI treated as a token. In this case, the input sequence represents a “set of brain regions” rather than a time series. This framework allows the model to directly learn the topological relationships between brain regions using self-attention mechanisms (Kan et al., 2022b).

Patch-based: Drawing inspiration from the Vision Transformer (ViT) proposed by Dosovitskiy et al. (2021), which introduced the idea of dividing 2D images into fixed-size patches for sequential input, this method adapts the concept to the fMRI domain. The 3D brain volume is directly divided into multiple non-overlapping 3D cubes (patches), and the voxel features within each patch are flattened and mapped to a single token. This method does not rely on manually defined brain atlases and is capable of preserving finer-grained local anatomical structures (Le et al., 2025).

#### 3.2.2 Advantages of non-Euclidean topology

Traditional convolutional neural networks (CNNs) have inherent architectural limitations when processing brain spatial data. CNNs rely heavily on local receptive fields and excel at processing regular Euclidean grid data. However, functional brain networks inherently exhibit highly non-Euclidean topological characteristics (Mohammadi and Karwowski, 2025)–brain regions that are functionally tightly coupled are often physically distant from one another (e.g., node interactions spanning the anterior and posterior regions of the brain in the default mode network) (Rahman et al., 2026).

Spatial Transformers overcome this limitation through self-attention mechanisms, demonstrating two key representational advantages. On the one hand, the spatial-dimensional attention matrix *A*∈ℝ^*N*×*N*^ can directly compute the correlation strength between any two locations in the entire brain. Mathematically, this is equivalent to constructing a learnable dynamic functional connectivity matrix, enabling the model to automatically discover latent brain network hubs in a fully data-driven manner, thereby reducing the reliance of traditional graph convolutional networks (GCNs) on predefined static graph structures (Wang L. et al., 2021). On the other hand, leveraging 3D positional embeddings, the Transformer can not only capture the brain's geometric folding patterns but also overcome the limitations imposed by physical anatomical distance, directly capturing physiologically meaningful long-range functional dependencies from a global perspective (Nguyen et al., 2025).

#### 3.2.3 Typical spatial transformers and application evaluation

Existing research has amply demonstrated the immense potential of spatial Transformers in overcoming the limitations of traditional local receptive fields and reconstructing the complex anatomical and topological structures of the entire brain (Han et al., 2024). Depending on the spatial scale at which tokens are constructed, their representative applications comprehensively cover multiple cutting-edge dimensions, ranging from macro-level brain network inference to micro-level voxel decoding.

In 3D representation and vision decoding tasks at the voxel and patch levels, spatial Transformers have demonstrated remarkable efficiency in processing massive amounts of redundant 3D data. Early indications of this potential came from 3D medical image segmentation models such as UNETR (Hatamizadeh et al., 2022), which demonstrated that purely Transformer-based architectures are fully capable of directly learning complex anatomical structures without explicit boundary guidance. In the field of fMRI, addressing the challenge that traditional methods often lose 3D spatial coordinates due to dimensionality reduction, Nguyen et al. proposed Brainformer, which innovatively introduced an attention mechanism that preserves the 3D absolute coordinates of brain regions. By explicitly modeling the collaborative patterns among voxels across the entire brain, it more accurately simulates the global cognitive logic of the human brain (Nguyen et al., 2025). In response to the massive computational overhead of 3D voxel data, VoxelFormer, proposed by Le et al., has emerged as a representative approach for large-scale spatial modeling. This model introduces a Token Merging (ToMer) strategy that enables the adaptive fusion of semantically similar spatial tokens in the deep layers of the network, preserving fine-grained features while significantly reducing computational complexity (Le et al., 2025). Furthermore, in the highly challenging task of cross-modal vision decoding, the Brain-IT model utilizes a brain-image interaction Transformer to extract high-order semantics from fMRI spatial signals, achieving high-quality natural image reconstruction and further expanding the application boundaries of spatial modeling (Beliy et al., 2026).

In the inference of complex network topologies at the region-of-interest (ROI) level, spatial Transformers are fundamentally reshaping the traditional graph convolutional network (GCN) paradigm. At this scale, each anatomical brain region is treated as an independent spatial token. BrainNetTF, proposed by Kan et al. (2022b), is a landmark work in this field. Figure 4 provides a conceptual illustration of the spatial Transformer paradigm, in which anatomical brain regions can be represented as spatial tokens and multi-head self-attention can be used to model non-Euclidean spatial interactions between brain regions. This fully data-driven approach to spatial topology inference effectively avoids information loss caused by subjectively defined graph network thresholds and has achieved significant progress in cross-subject brain atlas representation learning.

**Figure 4:**
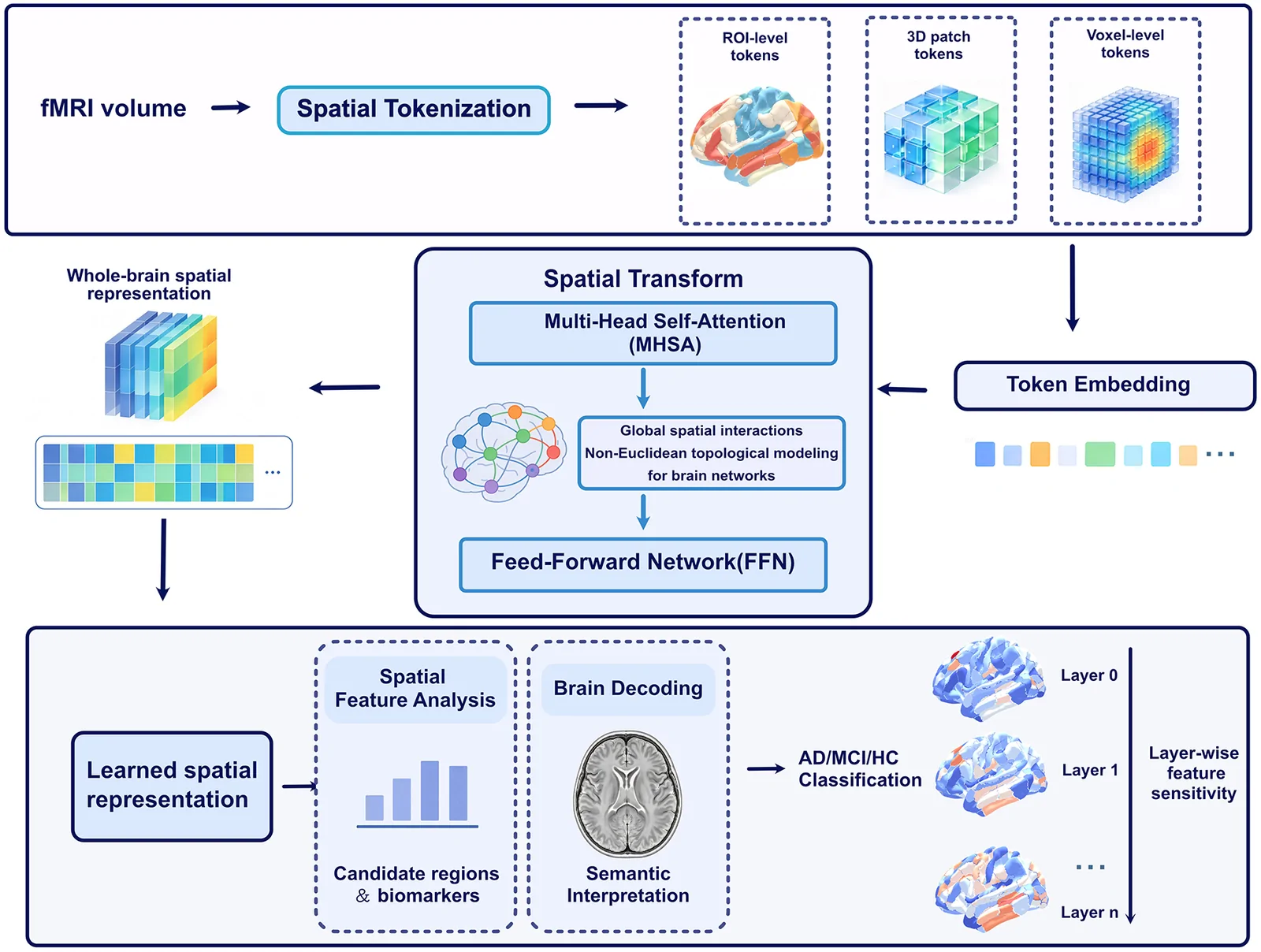
Conceptual overview of spatial Transformer modeling for fMRI analysis. Spatial tokenization can be performed at different spatial scales, including ROI-level, 3D patch-level, and voxel-level representations. Spatial Transformers then use multi-head self-attention to model global spatial interactions and non-Euclidean brain topology, supporting downstream spatial feature analysis and brain decoding.

In tasks involving spatial feature-driven clinical diagnosis and the discovery of potential biomarkers, the self-attention mechanism offers certain advantages in modeling whole-brain spatial dependencies, providing an effective tool for identifying key brain regions associated with diseases. The ASDFormer architecture proposed by Izadi and Safayani (2025) combines a Mixture of Experts (MoE) mechanism with a Transformer structure. By learning weighted representations of whole-brain spatial features, it can identify brain regions and functional connectivity patterns with high discriminative power in autism spectrum disorder (ASD) classification tasks. This method achieves adaptive modeling of whole-brain information without relying on explicit manual annotations and has demonstrated promising performance in ASD-related classification tasks, highlighting the potential of Transformers in assisting neuroimaging analysis and the exploration of potential biomarkers.

Although spatial Transformers excel at capturing the brain's topological structure, their primary limitation lies in inadequate modeling of temporal dynamics. Research indicates that functional connectivity in the brain is not static but exhibits significant fluctuations on the order of seconds and continuously reorganizes across different temporal scales (Vidaurre, 2024); BOLD signals themselves also embody complex stochastic dynamic processes (Scarciglia et al., 2024). However, existing methods often fail to fully integrate spatiotemporal dependencies, frequently simplifying the temporal dimension–a practice that has been shown to lead to the loss of critical representational information, thereby compromising performance in downstream tasks (Li et al., 2023). In fact, relying solely on spatial modeling makes it difficult to distinguish between cognitive states that share similar spatial distributions but exhibit markedly different temporal patterns. Capturing this dynamic reorganization across temporal scales is crucial for understanding cognitive flexibility and the pathological mechanisms underlying brain disorders (Ding et al., 2026).

### 3.3 Spatiotemporal Transformer modeling

Looking at the aforementioned temporal (Section 3.1) and spatial (Section 3.2) modeling paradigms, most adopt a “dimension-reduction and decoupling” strategy to mitigate the computational complexity of four-dimensional data. While this compromise improves engineering efficiency, it inevitably disrupts the brain's inherent spatiotemporal coupling: purely temporal modeling tends to lose fine-grained spatial topology, while purely spatial modeling often overlooks dynamic evolutionary processes.

However, network neuroscience suggests that brain function is essentially a dynamic system in which space and time are deeply intertwined (e.g., competitive switching between brain networks) (Watters et al., 2024). Forcing it into a single dimension makes it difficult to fully elucidate complex cognitive and pathological mechanisms (Xia et al., 2026). Therefore, “spatiotemporal joint modeling,” which simultaneously captures spatial dependencies and temporal dynamics within a unified framework, is emerging as one of the key evolutionary directions in fMRI-Transformer research.

#### 3.3.1 Decoupled and unified fusion strategies

For high-dimensional fMRI data of size *T*×*X*×*Y*×*Z*, existing research has weighed “computational feasibility” against “information completeness,” gradually giving rise to two representative modeling approaches: factorized spatiotemporal modeling strategies and unified modeling strategies.

Factorized Spatiotemporal Modeling involves a structural decomposition of temporal and spatial features, typically employing a cascaded architecture of “spatial modeling + temporal modeling,” in which intra-frame regional dependencies are first characterized, followed by the aggregation of dynamic information across time. Within this framework, Twin-Transformer models spatial dependencies and temporal dynamics through two parallel branches, effectively reducing spatiotemporal information interference and enhancing representational flexibility (Yu et al., 2022). Transformer3 further systematizes this approach by introducing three types of Transformers–Time, Region, and Batch–to model temporal correlations, spatial structures, and subject-specific variations, respectively. Its ablation experiments validate the importance of multidimensional modeling for predicting complex cognitive outcomes (Tian et al., 2025). Furthermore, STARFormer addresses the issue of short-term patterns being easily overlooked in long-range modeling through a dual-branch aggregation mechanism (Dong W. et al., 2025). Overall, these methods feature a clear structure, manageable computational overhead, and ease of adaptation to existing Transformer designs; however, phased modeling somewhat limits the ability to capture fine-grained spatiotemporal interactions.

Unified 4D Modeling treats fMRI as an integrated four-dimensional representation, performing attention computations directly in a 4D token space and thereby jointly modeling spatial neighborhoods and temporal continuity within a single framework. The representative method SwiFT (Figure 5) extends the shifted window mechanism of the Swin Transformer to 4D, achieving global information interaction through local window modeling and hierarchical shifting, thereby reducing computational complexity while maintaining the ability to perceive fine-grained structures (Kim et al., 2023). Building on this, 4DfCF introduces 4D cross-scale embeddings and a dual-path attention architecture to achieve collaborative modeling of local and global spatiotemporal dependencies, where 4D-SDA captures short-range interactions and 4D-LDA models long-range functional coupling, thereby balancing micro-level neural activity with macro-level network features (Zheng et al., 2026). Overall, such methods offer representational capabilities that more closely align with the spatiotemporal continuity of real brain activity, but they impose higher demands on computational resources and model design.

**Figure 5:**
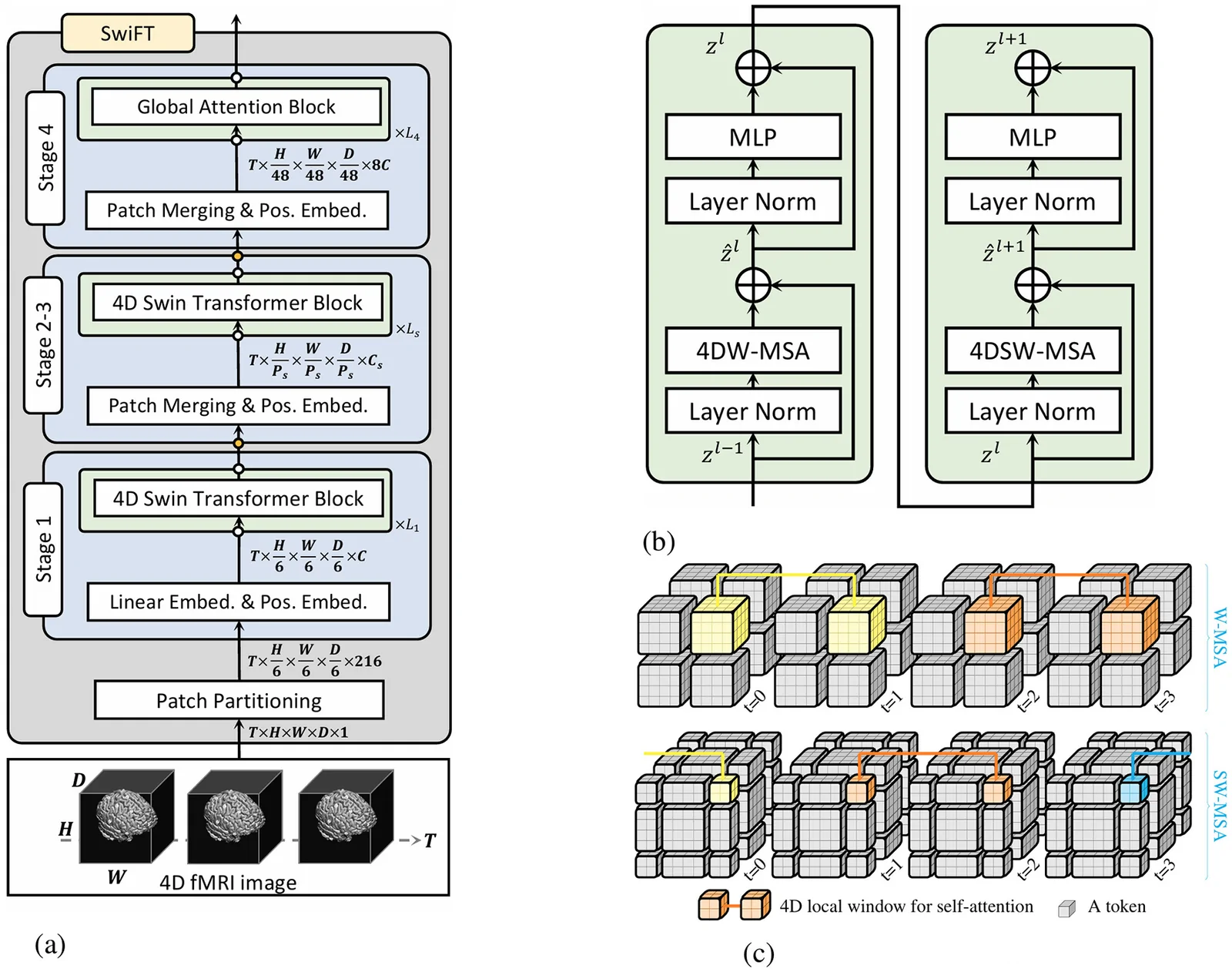
A typical spatiotemporal joint Transformer modeling architecture (SwiFT). This model employs a unified 4D fusion strategy to model directly on raw four-dimensional fMRI signals, capturing both spatial topology and temporal dynamics simultaneously through a 3D+1D hierarchical shifted window mechanism. Reproduced from Kim et al. (2023), from its arXiv preprint, licensed under CC BY 4.0. No modifications were made. **(a)** Overall architecture of SwiFT. **(b)** Successive 4D Swin Transformer blocks. **(c)** 4DW-MSA and 4DSW-MSA.

#### 3.3.2 Evolution of spatiotemporal tokens and modeling granularity

The core of spatiotemporal Transformer models lies in token design; the granularity of tokenization directly determines the level of neural activity the model can capture and its spatiotemporal fidelity. Existing research indicates a clear trend in spatiotemporal token design, evolving from “high-level abstraction” toward “fine-grained continuous modeling.”

In the early stages of macro-level abstraction, researchers typically employed a token construction method based on the “ROI × Time” framework. Specifically, this design spatially aggregates voxel-level BOLD signals within each brain region to obtain a single representative time series. While this spatial aggregation significantly reduces computational complexity and preserves coarse-grained temporal dynamics for characterizing interactions between brain regions, it inevitably obscures fine-grained spatial heterogeneity within the brain.

To overcome this limitation, modeling paradigms are increasingly moving toward native 4D voxel-level (voxel/patch-level) spatiotemporal joint modeling. For example, the recently proposed NeuroSTORM foundation model directly removes the constraints imposed by predefined atlases and employs a spatiotemporally interwoven voxel-level feature extraction mechanism within a whole-brain 4D space, enabling the model to capture large-scale, fine-grained brain dynamics across anatomical boundaries (Wang C. et al., 2026). Meanwhile, cutting-edge architectures such as MADCT-4D explicitly model the continuous temporal evolution of local brain voxel patches by integrating 4D spatiotemporal networks with dynamic functional connectivity sequences. Such designs, which construct continuous windows (4D Windows) or spatiotemporal primitives directly within the native high-dimensional spatiotemporal space, have been shown to enable Transformers to learn more refined spatiotemporal propagation patterns and enhance the sensitivity of deep models to the brain's complex dynamic functional connectivity (dFC) reconfiguration (Wang et al., 2026a).

#### 3.3.3 Generative and discriminative training paradigms

In addition to structural design, the choice of training objectives significantly influences a model's representational capacity and generalization boundaries. Current spatiotemporal models primarily fall into two main categories: discriminative and generative paradigms.

Discriminative frameworks are typically driven directly by downstream classification or prediction tasks, employing end-to-end supervised optimization and often exhibiting high sensitivity to features associated with specific clinical diagnoses. For example, the Multi-scale Spatio-Temporal Attention Network (MS-STAN) fits functional connectomes through supervised learning and has achieved strong classification performance in the identification of depression and autism (Kong et al., 2025). However, under “high-dimensional, small-sample” conditions, purely discriminative models may face an increased risk of overfitting to a single cohort and encounter difficulties in learning generalizable neural features across centers.

To address sample size limitations, generative paradigms have increasingly incorporated self-supervised learning objectives to learn intrinsic data distributions, with the goal of reconstructing the brain's spatiotemporal dynamics. For example, the Masked Momentum Contrastive Dynamic Transformer uses a self-supervised mechanism to reconstruct masked fMRI sequences, guiding the model to infer global spatiotemporal evolution patterns (Cheng et al., 2025a). This generative pre-training helps mitigate overfitting in low-sample-size scenarios and, to some extent, enhances the model's cross-domain robustness.

Overall, as the volume of brain imaging data continues to grow, purely discriminative approaches face certain challenges in terms of generalization. “Generative large-scale pre-training based on massive amounts of unlabeled data, combined with downstream discriminative fine-tuning,” is gradually emerging as a key trend in the academic community's exploration of general-purpose neuroimaging foundation models (Wang Y. et al., 2026).

#### 3.3.4 Explicit modeling of dynamic functional connectivity

The unique advantage of the spatiotemporal joint Transformer lies in its ability to explicitly model dynamic functional connectivity (dFC). Unlike static spatial models, which implicitly assume average connectivity, spatiotemporal joint models can capture the reorganization of brain networks over time, such as the dynamic switching between the default mode network and task-positive networks.

Zhang et al. (2025a) fully leveraged this advantage in their classification study of mild cognitive impairment (MCI), using a spatiotemporal Transformer to capture subtle dynamic interaction patterns within brain networks and introducing a contrastive learning strategy to enhance the model's robustness in small-sample scenarios. Meanwhile, the Brain Network State Transformer (BNST) framework proposed by Nie et al. (2025) further advanced this paradigm by explicitly modeling different brain state partitioning strategies through the representation of functional connectivity in a dynamic “state space.” Building on this foundation, researchers have begun to integrate deeper principles of neuroscience into such dynamic architectures, such as the recently proposed Core-Periphery Principle-guided State Space Model (CP-SSM). This method leverages biological constraints regarding “core brain regions (high connectivity, hub functions)” and “peripheral brain regions (supporting functions)” in brain networks, combining a State Space Model (SSM) architecture to classify functional connectivity patterns. It demonstrates that incorporating anatomical and functional organizational priors significantly enhances the model's ability to capture long-range spatiotemporal dependencies (Chen et al., 2025). Furthermore, addressing the issue that traditional methods often overlook higher-order network dependencies, the recently proposed LHDFormer successfully captures long-range, higher-order dynamic connections that evolve over time through a NeuroWalk strategy and a time-varying Transformer, thereby reconstructing the brain's complex communication mechanisms across multiple dimensions (Xue et al., 2025).

This capability is particularly crucial for analyses involving complex cognitive shifts or mental disorders, enabling the model not only to determine “whether an anomaly exists,” but also to reveal “when, where, and how the anomaly occurs.” This marks a significant step forward for spatiotemporal models, transforming them from “black-box classifiers” into “dynamic process analyzers.”

#### 3.3.5 Comparison of existing models and discussion of limitations

From the perspective of modeling capabilities, spatiotemporal Transformer models offer clear advantages over single-dimensional temporal or spatial paradigms in terms of information completeness, potential for biological interpretation, and data utilization efficiency. Time-based models tend to compress spatial structures, while space-based models often simplify or overlook temporal dynamics; in contrast, spatiotemporal joint modeling provides a unified framework for preserving and jointly modeling the four-dimensional information flow of fMRI data. However, its high computational complexity, reliance on large-scale data, and the limited interpretability of attention weights in high-dimensional scenarios remain urgent challenges. Despite the high computational cost, some studies have demonstrated that when combined with self-supervised pre-training and structural priors (such as brain segmentation or graph constraints), these models can achieve more robust generalization performance than single-dimensional models in small-sample scenarios (Gao et al., 2026), indicating their long-term potential for future neuroimaging modeling.

### 3.4 Self-supervised pre-trained transformer models

fMRI analysis has long been plagued by a structural dilemma between ultra-high-dimensional data and limited sample sizes (often referred to as the large *p*, small *n* problem). Due to high scanning costs, difficulties in recruiting participants, and the expertise-intensive nature of the annotation process, most studies typically have access to only a few hundred cases. However, fMRI data possess extremely high spatiotemporal dimensionality at the voxel level, with the feature dimensionality often reaching hundreds of thousands. This high-dimensional, small-sample characteristic makes purely supervised learning models highly prone to overfitting. Furthermore, differences in scanning equipment, acquisition protocols, and preprocessing procedures across multi-center datasets introduce significant multi-site domain shifts, further limiting model generalization in cross-site scenarios.

Against this backdrop, self-supervised learning (SSL) has gradually emerged as a key direction for the application of Transformers in fMRI research. This paradigm involves designing pretext tasks that do not require human annotation, leveraging large-scale unlabeled data to learn the intrinsic spatiotemporal patterns of brain activity, thereby providing more robust representation initialization for downstream tasks. This approach also lays the methodological foundation for constructing general-purpose “Brain Foundation Models.”

#### 3.4.1 Masked modeling for spatiotemporal representation learning

Masked modeling is one of the most widely used strategies in current self-supervised fMRI Transformers. The core idea is to randomly mask portions of the input signal in both spatial and temporal domains, prompting the model to reconstruct the missing content using the remaining contextual information. This forces the Transformer to learn the underlying latent generative distribution of the data and long-range dependency structures in neural activity.

Early research primarily focused on the temporal dimension. The TFF model proposed by Malkiel et al. (2022) applies random masking to ROI-level time series during the pre-training phase and requires the model to predict the masked time segments. In this way, the model is forced to capture contextual relationships across time points and brain regions. Experimental results show that this model significantly outperforms supervised models trained from scratch in disease recognition tasks such as schizophrenia, demonstrating particularly stable performance under small-sample conditions.

As research has progressed, masked modeling has gradually expanded from a single temporal dimension to a complete spatiotemporal structure. The 3D Masked Autoencoder (3D MAE) proposed by Gao et al. (2026) extends the masking strategy to 4D fMRI data by applying high-proportion random masking (e.g., 75%) to voxel blocks or spatiotemporal patches and requiring the model to reconstruct the complete signal. This challenging generative task effectively prevents the model from relying on simple strategies such as local interpolation, instead forcing it to learn the global constraints of brain anatomy and the cooperative organization patterns of functional networks. Experimental results demonstrate that representations obtained through this type of pre-training exhibit greater robustness in both few-shot tasks and cross-dataset transfer.

#### 3.4.2 Contrastive learning for individual difference modeling

Unlike masked modeling, which focuses on signal reconstruction, contrastive learning emphasizes the learning of discriminative representations through measures of similarity between samples. This approach typically enhances the model's sensitivity to individual differences by maximizing the consistency between positive sample pairs (such as different augmented views of the same subject) while simultaneously increasing the distance between negative sample pairs.

The SwiFUN framework proposed by Kwon et al. (2025) introduces contrastive learning mechanisms into fMRI cross-state prediction tasks. In the process of predicting task-specific activation maps from resting-state fMRI data, the model explicitly distinguishes subtle yet stable signal differences across participants through contrast-based analysis. This design significantly enhances the model's ability to capture individual specificity, enabling it to demonstrate strong generalization performance in cross-participant prediction tasks.

#### 3.4.3 Generalization of brain functional foundation models

With the continued growth in the volume of available unlabeled brain imaging data, Transformer-based “brain foundation models” have gradually emerged as a research focus. These models are typically pre-trained in a self-supervised manner on large-scale, multi-center datasets; their goal is not to optimize for a single task, but rather to learn highly transferable, general representations of brain activity.

As a representative work in this field, BrainLM (Ortega Caro et al., 2024) performs self-supervised pre-training on large-scale datasets—such as the UK Biobank, which contains approximately 80,000 scans—to construct a versatile foundation of pre-trained parameters. This paradigm not only supports zero-shot inference of brain latent spaces but also enables rapid fine-tuning using a small number of labeled samples, demonstrating significant advantages in few-shot learning for clinical prediction tasks such as depression and anxiety. Building on this, the BrainSymphony framework (Khajehnejad et al., 2025) further incorporates multimodal biological priors, utilizing Transformers to jointly model fMRI and structural connectivity (SC) during the pre-training phase, thereby aligning the representations more closely with the brain's true structure-function coupling patterns. Recent studies have also proposed a paradigm where fMRI data are projected onto a cortical unwrapped map and fed into a spatiotemporal MAE. For the first time, this approach validated on HCP data that model performance strictly follows a power scaling law, demonstrating that such foundation models exhibit greater robustness in cross-center scenarios (Lane et al., 2026).

Overall, self-supervised and pre-trained Transformers have driven the shift in fMRI modeling from a “task-driven” to a “representation-driven” approach. Through strategies such as masked reconstruction and contrastive learning, these models can learn spatiotemporal representations with biological structural constraints under unlabeled conditions and demonstrate superior generalization capabilities in small-sample and cross-center tasks. However, such representations are not necessarily neurobiologically interpretable. Ding et al. (2026) point out that while dynamic functional connectivity modeling holds promise, it still faces challenges such as insufficient statistical power, noise interference, and a lack of ground truth. Therefore, whether the features learned by pre-trained Transformers truly reflect the functional organization of the brain still requires validation through neuroscientific methods. In the future, hybrid pre-training strategies that integrate generative and discriminative objectives, alongside larger-scale, high-fidelity brain foundation models, are expected to further advance the development of general brain representation learning.

## 4 Transformers in fMRI applications

With continuous breakthroughs in the Transformer architecture's spatiotemporal modeling and representation learning capabilities, its application in fMRI research has gradually evolved from the stage of method feasibility validation toward the systematic exploration of real-world neuroscientific problems and clinical scenarios. This section provides a systematic review of representative advancements in Transformer applications within fMRI research across three dimensions: assisted diagnosis of brain diseases, learning of brain functional representations, and cross-subject and cross-site generalization capabilities. It also analyzes the advantages and limitations of Transformers relative to traditional deep learning methods.

### 4.1 Diagnosis and prediction of brain diseases

Intelligent diagnosis of brain disorders is one of the most critical clinical applications of fMRI analysis. Compared to traditional methods based on manual feature extraction or shallow models, Transformers can directly process high-dimensional spatiotemporal signals and automatically detect subtle functional connectivity abnormalities associated with disease pathology in complex settings involving multiple sites and large sample sizes. Currently, these methods have demonstrated significant advantages in diagnosing various brain disorders, including Alzheimer's disease (AD), major depressive disorder (MDD), and autism spectrum disorder (ASD).

For neurodegenerative diseases, capturing early, subtle patterns of functional network degeneration is a key challenge. Zhang et al. (2025a) proposed a dynamic functional connectivity modeling framework based on a spatiotemporal Transformer. By dividing fMRI time series into consecutive time windows using a sliding window and modeling the sequences with a self-attention mechanism along the temporal dimension, the framework more effectively captures the temporal dynamics associated with MCI and AD. Building on this, the 4DfCF model proposed by Zheng et al. (2026) further introduces a cross-scale attention mechanism, significantly enhancing the model's sensitivity to long-range functional connectivity disconnections in AD patients. To further leverage the complementary advantages of multimodal data, existing studies have constructed hybrid models that fuse convolutional and Transformer networks to perform multi-class Alzheimer's disease classification tasks. This method utilizes convolutional layers to extract fine-grained local features from images and relies on the Transformer's self-attention mechanism to uncover global multimodal correlations across the entire brain. Experiments demonstrate that this hybrid architecture can effectively distinguish between AD, MCI, and normal aging samples, exhibiting superior feature modeling capabilities and clinical generalization stability compared to single-network architectures (Alorf, 2025). Furthermore, to address the challenge of modeling functional connectivity networks, He et al. (2024) proposed a Spatiotemporal Graph Transformer Network (STGTN). By constructing a functional connectivity graph of brain regions from rs-fMRI data and utilizing a graph Transformer to simultaneously model both the spatial topological relationships and temporal dynamics between nodes, the method achieves automatic identification of Alzheimer's disease patients. Experimental results demonstrate that this method achieves superior performance compared to various traditional deep learning models in the classification task between AD and normal controls (NC), further validating the potential of Transformers to model complex neural connectivity patterns at the brain network level.

For psychiatric disorders lacking clear anatomical markers of pathology, Transformers have demonstrated strong capabilities in characterizing large-scale abnormalities in brain network coordination. In research on major depressive disorder (MDD), Dai et al. (2024) revealed abnormal functional connectivity patterns between the default mode network (DMN) and the visual network. In research on autism spectrum disorder (ASD), Wang et al. (2026a) proposed a Multi-Atlas Dynamic Connectivity Transformer (MADCT-4D), which models the time-varying characteristics of ASD-related brain functional networks by fusing 4D spatiotemporal features with dynamic functional connectivity information, achieving strong performance in classification tasks. Meanwhile, regarding attention-deficit/hyperactivity disorder (ADHD), Shi et al. (2025) utilized a Transformer to model whole-brain structural connectivity networks, revealing abnormal connectivity patterns involving the caudate nucleus and prefrontal functional networks, thereby confirming the critical role of structural topological alterations in the pathophysiological mechanisms of ADHD. Furthermore, in the diagnosis of schizophrenia (SCZ), recent research has proposed a Multi-Branch Cross-Attention Transformer (MBCAT). By spatially aligning and fusing the temporal functional features from fMRI with the sparse structural features from sMRI, and leveraging complementary cross-modal information, this approach has significantly improved the accuracy of identifying SCZ patients (Fan et al., 2024).

Furthermore, to address the challenge in clinical practice posed by the coexistence of multiple brain disorders with highly overlapping pathological features, Raza et al. (2025) proposed the NeuroMoE framework, which achieves accurate multi-class classification of complex neurological diseases (such as Parkinson's disease [PD] and its prodromal stage, iRBD) by introducing a Transformer-driven Mixture-of-Experts (MoE) mechanism. This model not only integrates multimodal imaging data–including fMRI, anatomical MRI (aMRI/sMRI), and diffusion tensor imaging (DTI)–along with clinical biomarkers, but also innovatively employs modality-specific experts and adaptive gating strategies to address the heterogeneity of multi-source data. While capturing complex spatial dependencies, this architecture effectively reduces the computational complexity of multimodal learning through sparse activation mechanisms, significantly improving the model's diagnostic accuracy and generalization performance in real-world clinical settings.

Overall, these studies indicate that Transformers not only improve the predictive accuracy of disease diagnosis but also provide interpretable insights into model decisions to some extent. However, as emphasized by (Farahani et al., 2022) in their systematic review on explainable AI in neuroimaging, “*post-hoc* explanations” that rely solely on model weights often lack causal validity; there remains a pressing need to develop built-in explainable architectures with stronger physiological constraints in the future. Furthermore, it should be noted that most existing studies still rely primarily on retrospective data and single-task settings; their stability, reproducibility, and ability to support individual-level diagnoses within real-world clinical decision-making processes remain to be further validated in larger-scale prospective studies.

### 4.2 Brain feature learning

In addition to directly addressing disease classification tasks, an increasing number of studies are exploring whether Transformers can learn brain functional representations that are neurobiologically meaningful. The central question in this area is whether the information encoded in the model's latent space exhibits stability, transferability, and potential biological interpretability.

In terms of predicting individual characteristics and cognitive abilities, the Transformer3 framework proposed by Tian et al. (2025) demonstrates the potential of purely Transformer-based architectures for learning brain functional representations. By employing independent temporal, spatial, and subject-level Transformers, this model systematically decouples and models the multidimensional structure of fMRI data. Experimental results indicate that the learned representations effectively support the prediction of individual characteristics such as fluid intelligence, age, and gender, and exhibit strong transferability across different downstream tasks. Sun et al. (2025a) further applied the Transformer to brain state recognition, achieving effective differentiation of various brain functional states through deep modeling of fMRI spatiotemporal patterns. To capture state representations at finer spatial scales, recent research has introduced the Swin Transformer architecture to directly process voxel-level data. Through a hierarchical shifted-window mechanism, this model effectively overcomes the quadratic computational complexity bottleneck posed by high-resolution fMRI, significantly enhancing the accuracy of brain state prediction while preserving micro-local activation features (Sun et al., 2025b).

In higher-level cognitive and semantic modeling tasks, the Transformer's powerful representation learning and cross-modal alignment capabilities have been increasingly applied to neural encoding and decoding research. Recent reviews indicate that neural encoding and decoding, particularly for visual information, aims to establish a precise mapping between the feature space of external stimuli and the space of brain activity. The core challenge lies in how to leverage deep architectures to capture the highly complex, high-dimensional, and nonlinear neural representation processes involved in the brain's processing of sensory information (Liu et al., 2025). Against this backdrop, the Transformer architecture, leveraging its global modeling capabilities, has emerged as a key technology for bridging neural signals and complex semantic features. For example, Brainformer, proposed by Nguyen et al. (2025), incorporates a hierarchical architecture inspired by the processing mechanisms of the visual cortex to achieve a mapping from fMRI signals to the visual feature space; Le et al. (2025)'s VoxelFormer, on the other hand, employs a Token Merging strategy to achieve parameter-efficient multi-subject visual decoding while reducing computational complexity. In this field, the MinD-Vis model proposed by Chen et al. (2023) represents a landmark achievement. This study combines a masked brain model with a dual-conditional latent diffusion model (DC-LDM) to generate photorealistic images with high semantic and detail fidelity from fMRI signals using only a limited amount of paired labeled data, significantly surpassing state-of-the-art (SOTA) performance in visual decoding. Furthermore, the Brain-IT framework proposed by Beliy et al. (2026) explicitly models dynamic interactions between brain regions via a Brain-Interaction Transformer, significantly improving the fidelity and semantic consistency of visual image reconstruction from fMRI signals. Meanwhile, Tang et al. (2023) demonstrated that brain encoding models based on multimodal Transformers can achieve cross-modal transfer between visual and natural language representations. The latest Neuro-Vision model has even achieved end-to-end generation from brain signals to images and then to natural language descriptions (Shen et al., 2024), marking a broader shift in fMRI research from low-level feature extraction toward higher-level semantic understanding and cross-modal generation.

Beyond decoding visual or semantic information from brain signals, recent studies have also begun to explore the *text-to-brain encoding* task, which aims to predict patterns of brain activity from textual semantic information. Its core objective is to establish mappings between neuroscientific text and spatial brain representations. For example, Text2Brain employs a Transformer-based neural language model to extract semantic information from free-form text queries and subsequently generate corresponding brain activation maps, enabling the prediction of spatial brain representations from neuroscientific text (Ngo et al., 2022). In addition, NeuroConText investigates cross-modal alignment between neuroscientific text and brain activity representations through contrastive learning, incorporating rich semantic representations of neuroscience literature to support text-to-brain retrieval and brain map reconstruction (Ghayem et al., 2026). These studies suggest that the cross-modal modeling capabilities of Transformer architectures can be applied not only to decode external stimuli or semantic information from brain signals, but also to leverage semantic knowledge contained in text for the prediction and retrieval of brain activity patterns. Looking ahead, a promising direction for further exploration is to jointly leverage pretrained language models and Transformer-based brain imaging foundation models to investigate cross-modal alignment between textual and brain representations. For instance, compared with conventional predefined atlas-based representations, future studies may further explore the use of high-dimensional feature embeddings learned by pretrained brain models as representations of brain activity or neuroimaging data, thereby providing new modeling perspectives for multimodal neuroimaging meta-analysis, brain activity encoding, and cross-modal retrieval tasks.

It is important to emphasize that, although the aforementioned studies have made significant progress in terms of downstream task performance and representation transferability, whether the latent spaces learned by the models truly correspond to interpretable neural computational mechanisms still requires more rigorous verification through a combination of neuroscientific experimental paradigms and statistical analysis methods. Therefore, improvements in representation performance do not necessarily equate to a direct characterization of neural mechanisms, and this issue remains a major challenge in current research.

### 4.3 Cross-subject and cross-site generalization

Cross-subject and cross-site generalization capabilities are among the key bottlenecks limiting the clinical translation of intelligent fMRI analysis models. Individual differences among subjects, as well as inconsistencies in equipment, parameters, and protocols across scanning sites, often introduce significant non-biological noise, leading to a substantial decline in model performance on external datasets.

The potential advantage of Transformers in this context stems from their ability to model the system globally. Compared to convolutional networks that rely on local receptive fields, self-attention mechanisms can integrate spatiotemporal information on a global scale, thereby making it more likely to learn abstract features independent of specific scanning conditions. Based on this idea, Brain Foundation Models such as BrainLM have effectively improved the model's robustness to scanner bias through masked self-supervised pre-training on large-scale, multi-site datasets (Ortega Caro et al., 2024).

In addition to leveraging large-scale pre-training, incorporating multimodal metadata for collaborative modeling has also emerged as an effective approach to enhancing generalization capabilities. The multimodal Transformer framework proposed by Jazi and Hajiesmaeili (2025) achieves more robust brain state decoding by integrating fMRI time series with medical metadata, such as age, gender, and clinical indicators. This study demonstrates that medical metadata can provide important contextual information for high-noise imaging signals. By leveraging the Transformer's cross-attention mechanism to capture the complex interactions between imaging features and clinical characteristics, the model significantly improves classification accuracy and generalization performance across multi-center and multi-task scenarios.

Furthermore, to address the generalization challenges posed by inter-subject individual differences, the SwiFUN model proposed by Kwon et al. captures individual-specific information latent in resting-state dynamics through a contrastive learning mechanism, thereby maintaining stable predictive performance on unseen subjects (Kwon et al., 2025). To more thoroughly address non-biological variability across centers, the Graph Disentanglement Learning framework uses Transformers and GNNs to explicitly disentangle disease features, clinical covariates (such as site and age), and individual variability features from fMRI signals, achieving strong generalization performance across multiple multi-center datasets (Zhang et al., 2025c). Nevertheless, Transformers cannot fundamentally eliminate domain shift issues. Current research typically still requires combining data standardization, feature alignment, or explicit domain adaptation strategies to achieve reliable performance in real-world multicenter clinical settings (Abraham et al., 2014). Notably, a recent 4D fMRI conditional generative model based on the Diffusion Transformer (DiT) can directly synthesize realistic spatiotemporal signals, providing a potential new direction for addressing the challenges of small sample sizes and domain shift through virtual experiments and cross-center data harmonization (Seo et al., 2025).

## 5 Comparative analysis and comprehensive discussion

Having systematically reviewed the main technical approaches of Transformers in fMRI research, it is necessary to conduct a comprehensive analysis of the differences between various methods and their applicability from a broader perspective. With the widespread adoption of the Transformer architecture, its advantages over traditional methods have been validated in numerous empirical studies. However, model selection should not rely solely on improvements in performance metrics; there is always a trade-off between modeling paradigms in terms of expressive power, computational tractability, and applicable scenarios. This section provides a systematic analysis across three dimensions: differences among modeling paradigms, comparisons between Transformers and other deep learning models, and discussions on interpretability and neuroscientific validity.

### 5.1 Comparison of transformer modeling paradigms

Based on the classification framework proposed in Section 3, current fMRI-based Transformer methods can be broadly categorized into three types: temporal (TS-wise), spatial (Space-wise), and spatiotemporal. These paradigms differ substantially in their tokenization strategies, inductive biases, information retention, computational costs, and applicable scenarios. A detailed comparison is provided in Table 1.

**Table 1:** Comparison of transformer modeling paradigms for fMRI analysis.

Modeling paradigm	Representative models	Tokenization strategy	Inductive biases	Information retention and attenuation	Computational cost and applicable scenarios
Temporal	VTFF (Wang, 2025) BolT (Bedel et al., 2023) TS-Transformer (Zhou et al., 2025)	ROI spatial aggregation → temporal tokens	ROI-level spatial homogeneity Cross-time dependencies	Retained: Macroscopic temporal dynamics Attenuated: Fine-grained spatial details within ROIs	Cost: *O*(*T*^2^) Scenarios: Cognitive decline and MDD classification
Spatial	BrainNetTF (Kan et al., 2022b) Brainformer (Nguyen et al., 2025) KAGT (Song et al., 2025)	ROI/brain-region representations → spatial tokens or graph nodes	Topological relationships between brain regions Functional connectivity or structural priors	Retained: Spatial relationships and network topology Limited: Explicit temporal dynamics	Cost: $O\left(N_{\text{roi}}^{2}\right)$ Scenarios: Brain network analysis and cross-center diagnosis
Spatio-temporal joint	SwiFT (Kim et al., 2023) 4DfCF (Zheng et al., 2026) STGTN (He et al., 2024)	ROI/voxel/patch representations → spatiotemporal tokens	Local spatiotemporal continuity Joint spatial-temporal dependencies	Retained: Fine-grained spatiotemporal dynamics Potential attenuation: Patchification/downsampling details	Cost: Relatively high Scenarios: Complex brain dynamics and large-scale pre-training

*N*_roi_ denotes the number of brain regions of interest, and *T* denotes the number of time points. Computational costs are approximate and may vary depending on the specific attention mechanism, tokenization strategy, and model architecture.

From a methodological perspective, temporal modeling approaches (such as VTFF) typically represent fMRI data as multivariate time series and utilize self-attention mechanisms to capture dynamic patterns over long timescales. By primarily focusing on dependencies across time points, these methods are well suited for analyzing disease-related global functional fluctuations. However, since they generally rely on ROI-level signal representations, their ability to characterize fine-grained spatial heterogeneity within brain regions is relatively limited (Wang, 2025). In contrast, spatial modeling methods (e.g., Brainformer) characterize relationships among brain regions by constructing spatial tokens or graph-based representations. By emphasizing topological relationships and structural or functional connectivity patterns between brain regions, these methods are more suitable for preserving large-scale spatial network organization, although their ability to characterize rapidly evolving temporal dynamics may be relatively limited (Nguyen et al., 2025). Spatiotemporal joint modeling, on the other hand, jointly models spatial and temporal information through ROI-, voxel-, or patch-level representations, thereby providing a more comprehensive characterization of brain activity. These methods can generally preserve finer-grained spatiotemporal dynamics, although some local details may still be attenuated during patchification or downsampling. However, modeling high-dimensional spatiotemporal signals often incurs substantial computational and memory overhead, particularly when global self-attention mechanisms are employed. To alleviate this issue, recent studies have adopted strategies such as local windowing or hierarchical modeling. For instance, the Spherical Spatio-Temporal Transformer (STF) decouples attention computations across “macro-tiles” and “micro-nodes,” maintaining computational tractability while preserving the ability to model global spatiotemporal dependencies (Cheng et al., 2025b).

### 5.2 Comparison with other deep learning models

The rise of Transformers in fMRI research stems in part from the alignment between their global modeling capabilities and the distributed nature of brain functional networks. However, different deep learning architectures exhibit distinct characteristics in terms of inductive biases, data requirements, and generalization behavior. Therefore, Transformers do not possess an absolute advantage in all application scenarios, while traditional deep learning models remain suitable under specific data scales and task conditions.

Compared with convolutional neural networks (CNNs), Transformers do not rely on fixed local receptive fields; instead, they establish global dependencies through self-attention, making them more suitable for capturing functional coupling between distant brain regions. CNN-based methods, represented by BrainNetCNN, offer high parameter efficiency in extracting local features and benefit from relatively strong inductive biases, making them generally easier to train effectively when the amount of training data is relatively limited. However, their fixed convolutional structures may lack sufficient flexibility for modeling complex non-Euclidean brain networks and long-range dependencies (Kawahara et al., 2017). Shamshad et al. (2023) pointed out that the global self-attention mechanism of Transformers provides greater flexibility in modeling complex anatomical relationships. For example, the TransBTS study demonstrated that incorporating Transformers into a convolutional network architecture can enhance the modeling of long-range dependencies and global contextual information, thereby improving performance on complex medical image segmentation tasks (Wang W. et al., 2021). Therefore, hybrid architectures such as MedViT (Manzari et al., 2023) combine the advantages of CNNs in local feature extraction with the capability of Transformers to model global relationships.

Compared to Recurrent Neural Networks (RNNs), Transformers offer significant advantages in parallel computation and long-term sequence modeling. While RNNs/LSTMs are traditional choices for fMRI time-series and can be relatively efficient for moderate-sized datasets, their sequential structure limits parallelization and may make learning long-range dependencies more challenging in long sequences (Wang et al., 2020). Transformers directly model dependencies between arbitrary positions, enabling more flexible modeling of long-range functional patterns across temporal scales (Huang et al., 2026). However, this flexibility generally comes with weaker sequential inductive biases and potentially higher data requirements.

Compared to Graph Neural Networks (GCNs/GNNs), Transformers provide a more flexible mechanism for modeling global dependencies. Traditional graph-based approaches often rely on predefined or explicitly constructed connectivity structures, which provide strong topological priors and can be advantageous when training data are limited (Wang L. et al., 2021). However, the performance and generalization of such models may be influenced by variations in graph construction or connectivity estimation across datasets and centers. In contrast, the self-attention mechanism of Transformers can adaptively estimate relationships between tokens based on the input data, providing greater flexibility for modeling dynamic functional connectivity patterns (Asadi et al., 2023). Nevertheless, this reduced reliance on predefined structural priors may also increase the dependence of Transformer models on training data and appropriate regularization. Table 2 summarizes these differences in inductive biases, data requirements, generalization characteristics, and methodological advantages and limitations.

**Table 2:** Comparison of Transformers and mainstream deep learning models for fMRI modeling.

Model	Literature	Core mechanisms and inductive biases	Data requirements	Generalization	Pros and cons
CNN	BrainNetCNN (Kawahara et al., 2017) TransBTS (Wang W. et al., 2021)	Local receptive fields and translation invariance Emphasizes local spatial correlations	Moderate Suitable for small- to medium-scale datasets	Limited Less adaptable to topological variations	Pros: Efficient local feature extraction Cons: Limited ability to model long-range dependencies
RNN/LSTM	ST-NET (Wang et al., 2020)	Sequential recurrence and temporal continuity Emphasizes dependencies between adjacent time points	Moderate Relatively low parameter requirements	Limited Long-sequence modeling remains challenging	Pros: Effective for sequential modeling Cons: Lacks explicit spatial modeling
GCN/GNN	Graph-CNN (Wang L. et al., 2021)	Graph structure and message passing Relies on predefined or learned topological priors	Low to moderate Suitable for small-sample brain network data	Sensitive to graph structure Performance may be affected by graph shifts across centers	Pros: Well suited for non-Euclidean brain networks Cons: Dependent on graph construction
Transformer	Transformer3 (Tian et al., 2025) ST-Transformer (Asadi et al., 2023)	Global self-attention with relatively weak inductive biases Learns long-range dependencies adaptively	High Often benefits from large datasets or pre-training	Strong potential Suitable for transfer and cross-domain applications	Pros: Effective global and long-range dependency modeling Cons: High data and computational demands; prone to overfitting

Data requirements and generalization ability are relative assessments and may vary depending on the specific task, dataset size, model design, and training strategy.

### 5.3 Interpretability and neuroscientific validity

Despite their performance, the interpretability of Transformers remains a central point of contention. A critical question is whether self-attention weights can be directly mapped to neuroscientific functional connectivity.

Jain and Wallace (2019) argued that attention weights do not necessarily reflect the true contribution of input features and can be perturbed without affecting outputs. However, Wiegreffe and Pinter (2019) refuted this, demonstrating that under specific architectures, attention weights provide consistent insights into internal decision-making. This debate suggests that in fMRI, interpreting attention maps as pathogenic mechanisms carries methodological risks and should not be equated with physiological causality.

Different interpretability techniques target different objects. Attention weights reflect internal feature interaction patterns, whereas gradient- or perturbation-based methods focus on the sensitivity of model outputs to specific brain regions or time windows. Thus, the reliability of results depends on whether the interpretability level aligns with the research question. To enhance neuroscientific credibility, researchers have begun combining methods such as Grad-CAM and SHAP to assess the marginal impact of input features (Farahani et al., 2022).

Based on these considerations, the discussion of Transformer interpretability can be further extended by comparing Transformers with other established fMRI modeling approaches. Mature linear latent-variable methods, such as Independent Component Analysis (ICA) and Dictionary Learning, remain valuable in current fMRI analysis. Their relatively well-defined mathematical structures and more direct interpretation pathways enable them to characterize brain activity patterns from different perspectives and facilitate the analysis of subject-specific variability (Calhoun et al., 2009; Jain et al., 2023). For example, ICA can extract latent brain networks with spatial and temporal characteristics from fMRI data and has been widely used to characterize brain activity patterns (Calhoun et al., 2009). More recent studies have combined ICA-derived functional network connectivity features with dictionary learning to identify interpretable patterns and discriminative features from resting-state fMRI data (Ghayem et al., 2023). In contrast, Transformer models can capture complex nonlinear relationships and long-range dependencies in fMRI signals through self-attention mechanisms, but the interpretation of their internal representations remains challenging. Therefore, future research could explore how to incorporate the interpretability advantages of traditional linear latent-variable methods and further investigate their complementarity or integration with Transformer architectures, potentially providing new approaches for improving the neuroscientific validity and clinical utility of fMRI models.

In summary, Transformers offer a powerful global framework for fMRI, though they face challenges in complexity and interpretability. Future research must balance expressive power, efficiency, and neuroscientific validity to further their clinical application.

## 6 Challenges and future directions

Although the Transformer architecture has demonstrated significant potential in fMRI spatiotemporal modeling, feature learning, and cross-center generalization, its transition from methodological research to stable practical application still faces multiple constraints. Overall, these issues primarily manifest in terms of data scale, computational efficiency, and biological interpretability, and are to some extent intertwined.

First, the “high-dimensional, small-sample” nature of fMRI data remains a significant factor limiting model performance. A single subject typically comprises hundreds of thousands of voxels and hundreds of time points, yet due to acquisition costs and ethical constraints, the available sample size is often limited to just a few thousand. This data distribution makes large-scale Transformers prone to overfitting during training and poses challenges for cross-dataset generalization. For example, although BrainLM was pre-trained on large-scale scan data, its performance remains limited in certain downstream tasks with small sample sizes (such as PTSD) (Ortega Caro et al., 2024); methods like VoxelFormer have mitigated this issue to some extent through parameter-efficient fine-tuning, but the fundamental trade-off between data scale and model complexity remains unresolved (Le et al., 2025).

Second, from a computational perspective, the complexity introduced by whole-brain 4D modeling also poses practical constraints. If self-attention is computed directly in the joint voxel-time space, its computational complexity grows quadratically with the scale of the data. Although methods such as SwiFT approximate and optimize computations through local windowing mechanisms, thereby reducing computational overhead to some extent, such strategies typically involve trade-offs in the ability to model long-range dependencies (Kim et al., 2023). Therefore, effectively addressing the high demands on video memory and computational power posed by 4D data while ensuring high-precision feature extraction remains one of the challenges facing current models as they move toward clinical deployment.

In addition, issues regarding model interpretability and biological plausibility have gradually garnered attention. Previous studies have shown that attention weights do not necessarily directly reflect feature importance, and simply treating them as an explanation for functional connectivity may introduce bias (Jain and Wallace, 2019). At the same time, most current models remain primarily data-driven and lack explicit constraints regarding anatomical structures or physiological mechanisms, which to some extent limits their interpretive value in neuroscience research (Cong et al., 2024).

Although Transformers have demonstrated strong spatiotemporal representation capabilities in fMRI analysis, several unresolved challenges remain in its practical applications. First, there is a certain mismatch between the relatively large model capacity of Transformer and the limited number of independent samples available in current fMRI datasets. Although individual fMRI time series typically contain a large number of temporal samples, in most disease classification tasks, statistically independent samples are primarily defined at the subject level. The limited number of subjects may lead to overfitting in complex Transformer models and constrain their generalization performance across datasets, imaging centers, and populations. Second, although global self-attention can capture dependencies between distant brain regions, the statistical associations learned by the model are not necessarily equivalent to actual anatomical connections or physiological interactions. How to effectively incorporate brain-region topology, structural connectivity, and neurophysiological mechanisms into the representation learning process of Transformer therefore remains an open problem. Third, as the number of time points, brain regions, or voxels increases, the computational and memory costs of global self-attention grow substantially with the number of tokens. Existing window-based, sparse, and hierarchical attention strategies can reduce the computational burden, but they generally require a trade-off between the ability to model global dependencies and computational efficiency. This issue is also reflected in different Transformer-based fMRI modeling paradigms: temporal, spatial, and spatiotemporal joint modeling emphasize different aspects of information representation and require trade-offs among spatial details, temporal dynamics, and computational cost. A unified modeling framework that can simultaneously achieve high-quality spatiotemporal representation, computational efficiency, and biological plausibility remains lacking. Finally, attention weights in Transformer do not have a simple one-to-one correspondence with feature importance, functional connectivity, or underlying neural mechanisms. How to distinguish the statistical associations relied upon by the model for prediction from brain-region interactions with genuine neurobiological significance remains an open issue, and a unified and reliable evaluation standard is still lacking. Furthermore, research on brain foundation models further amplifies these challenges: fMRI datasets from different sources exhibit substantial heterogeneity in scanning parameters, subject populations, data quality, and preprocessing procedures. Establishing unified data standards, pretraining tasks, and evaluation protocols therefore remains an important challenge for developing general-purpose brain foundation models with robust cross-dataset generalization.

Against this backdrop, relevant research is gradually exploring modeling approaches with greater structural constraints and generalization capabilities. For example, incorporating neuroscientific prior knowledge (such as structural connectivity or anatomical pathways) to guide attention mechanisms has been shown to enhance model stability and interpretability (Wei et al., 2024). At the same time, multimodal information fusion is also considered a key direction; by integrating fMRI, structural images, and even behavioral or linguistic signals, it is expected to enable the construction of more comprehensive representations of brain function (Tang et al., 2023; Shen et al., 2024; Sheng et al., 2021; Wang W. et al., 2021). At the model level, self-supervised pretraining based on large-scale data is driving the development of foundational models for brain function. For example, recent research has successfully constructed a foundational model capable of generalizing to various neural phenotype prediction tasks by introducing a self-supervised masked training paradigm, enabling the network to learn neural dynamic features with greater generality and transferability (He et al., 2025).

On the other hand, research on model interpretability is also gradually deepening. In addition to attention visualization, methods based on perturbations or attribution (such as SHAP and LIME) are used to quantify the contributions of different brain regions to prediction outcomes, thereby providing a more verifiable analytical basis (Farahani et al., 2022). In practical applications, these methods have been used to identify key brain regions associated with diseases, and the results align to some extent with existing neuroscientific findings (Dai et al., 2024). Overall, how to enhance a model's interpretability and biological consistency while maintaining its expressive power remains a topic worthy of continued attention in the future.

## 7 Conclusion

This paper systematically reviews the theoretical foundations, modeling paradigms, and cutting-edge applications of the Transformer architecture in fMRI analysis. Looking back at the evolution of fMRI analysis methods, we can clearly observe a paradigm shift from “local, static, and small-sample” approaches to “global, dynamic, and foundational models.” The introduction of Transformers is not only aimed at achieving higher prediction accuracy but also at addressing the inductive bias limitations of traditional deep learning models (such as CNNs and RNNs) when handling non-Euclidean brain networks and long-term cognitive states.

First, the classification framework proposed in this paper–TS-wise, Space-wise, and spatiotemporal modeling–provides a structured perspective for understanding the existing literature. Empirical studies indicate that each of these three paradigms has its own strengths: TS-wise models (such as VTFF) excel at capturing early temporal fluctuations in neurodegenerative diseases; Space-wise models (e.g., Brainformer) demonstrate strong capabilities in aligning brain representations with visual semantic features in visual semantic decoding tasks; and spatiotemporal models (e.g., SwiFT, 4DfCF) provide a more comprehensive framework for characterizing complex brain network reorganization by explicitly modeling 4D dynamics.

Second, the rise of self-supervised learning and “brain foundation models” marks the entry of fMRI research into the era of large-scale models. Through masked pre-training on large-scale unlabeled data, these models can mitigate the effects of small sample sizes and cross-center variations, thereby learning brain functional representations with strong generalization capabilities. Such representations not only enhance the models' transferability across different data distributions but also enable Transformers to evolve beyond specific tasks into a general-purpose modeling framework capable of adapting to a wide range of downstream tasks.

Finally, despite challenges such as high computational complexity and debates over interpretability, the Transformer has undeniably become a key bridge connecting artificial intelligence and neuroscience. Not only has it improved the level of auxiliary diagnosis for brain disorders such as depression and Alzheimer's disease (Dai et al., 2024), but through integration with multimodal large models (such as Neuro-Vision), it has also enabled us to decode the brain's semantic space with unprecedented precision (Shen et al., 2024). In the future, with the introduction of structured prior knowledge and the maturation of causal inference techniques, Transformers are expected to help us decipher the neural coding mechanisms underlying brain function, propelling computational neuroscience toward the ultimate goal of “general brain decoding.”

## References

[B1] Abraham A. Pedregosa F. Eickenberg M. Gervais P. Mueller A. Kossaifi J. . (2014). Machine learning for neuroimaging with scikit-learn. Front. Neuroinform. 8:14. doi: 10.3389/fninf.2014.00014 PMC3930868 24600388

[B2] Abrol A. Fu Z. Salman M. Silva R. Du Y. Plis S. . (2021). Deep learning encodes robust discriminative neuroimaging representations to outperform standard machine learning. Nat. Commun. 12:353. doi: 10.1038/s41467-020-20655-6 PMC7806588 33441557

[B3] Alorf A. (2025). Transformer and convolutional neural network: A hybrid model for multimodal data in multiclass classification of alzheimer's disease. Mathematics 13:1548. doi: 10.3390/math13101548

[B4] Asadi N. Olson I. R. Obradovic Z. (2023). A transformer model for learning spatiotemporal contextual representation in fMRI data. Netw. Neurosci. 7, 22–47. doi: 10.1162/netn_a_00281 PMC10270708 37334006

[B5] Avberšek L. K. Repovš G. (2022). Deep learning in neuroimaging data analysis: Applications, challenges, and solutions. Front. Neuroimag. 1:981642. doi: 10.3389/fnimg.2022.981642 PMC10406264 37555142

[B6] Bai S. Kolter J. Z. Koltun V. (2018). An empirical evaluation of generic convolutional and recurrent networks for sequence modeling. arXiv [preprint] arXiv:1803.01271. doi: 10.48550/arXiv.1803.01271

[B7] Bannadabhavi A. Lee S. Deng W. Ying R. Li X. (2023). “Community-aware transformer for autism prediction in fMRI connectome,” in Medical Image Computing and Computer Assisted Intervention-MICCAI 2023, eds. Greenspan H. Madabhushi A. Mousavi P. Salcudean S. Duncan J. Syeda-Mahmood T. Taylor R. (Cham: Springer Nature Switzerland), 287–297.

[B8] Bedel H. A. Sivgin I. Dalmaz O. Dar S. U. Çukur T. (2023). BolT: Fused window transformers for fMRI time series analysis. Med. Image Anal. 88:102841. doi: 10.1016/j.media.2023.102841 37224718

[B9] Beliy R. Zalcher A. Jonathan Kogman J. Wasserman N. Irani M. (2026). “Brain-IT: image reconstruction from fMRI via Brain-Interaction Transformer,” in The Fourteenth International Conference on Learning Representations. Available online at: https://openreview.net/forum?id=9KjXqkfbPw

[B10] Bessadok A. Mahjoub M. A. Rekik I. (2023). Graph neural networks in network neuroscience. IEEE Trans. Pattern Anal. Mach. Intell. 45, 5833–5848. doi: 10.1109/TPAMI.2022.3209686 36155474

[B11] Bhavna K. Akhter A. Banerjee R. Roy D. (2024). Explainable deep-learning framework: decoding brain states and prediction of individual performance in false-belief task at early childhood stage. Front. Neuroinform. 18:1392661. doi: 10.3389/fninf.2024.1392661 PMC11239353 39006894

[B12] Calhoun V. D. Liu J. Adali T. (2009). A review of group ICA for fMRI data and ICA for joint inference of imaging, genetic, and ERP data. Neuroimage 45, S163–172. doi: 10.1016/j.neuroimage.2008.10.057 PMC2651152 19059344

[B13] Chen J. Lu Y. Yu Q. Luo X. Adeli E. Wang Y. . (2021). TransUNet: Transformers make strong encoders for medical image segmentation. arXiv [preprint] arXiv:2102.04306. doi: 10.48550/arXiv.2102.04306

[B14] Chen M. Yu X. Zhang J. Chen T. Cao C. Zhuang Y. . (2025). “Core-periphery principle guided state space model for functional connectome classification,” in Proceedings of Medical Image Computing and Computer Assisted Intervention-MICCAI 2025, volume LNCS 15971 (Cham: Springer Nature Switzerland), 236–246. 10.1007/978-3-032-05162-2_23 PMC12715851 41425911

[B15] Chen X. Wang X. Zhang K. Fung K.-M. Thai T. C. Moore K. . (2022). Recent advances and clinical applications of deep learning in medical image analysis. Med. Image Anal. 79:102444. doi: 10.1016/j.media.2022.102444 PMC9156578 35472844

[B16] Chen Z. Qing J. Xiang T. Yue W. L. Zhou J. H. (2023). “Seeing beyond the brain: Conditional diffusion model with sparse masked modeling for vision decoding,” in 2023 IEEE/CVF Conference on Computer Vision and Pattern Recognition (CVPR) (Vancouver, BC: IEEE), 22710–22720.

[B17] Cheng J. Hu D. Wu Z. Yuan X. Li G. (2025a). “Masked momentum contrastive dynamic transformer for self-supervised functional connectivity representation learning,” in 2025 IEEE 22nd International Symposium on Biomedical Imaging (ISBI) (Houston, TX: IEEE), 1–5. 10.1109/isbi60581.2025.10980976 PMC12490092 41050554

[B18] Cheng J. Zhao F. Wu Z. Yuan X. Wang L. Gilmore J. H. . (2025b). STF: A spherical transformer for versatile cortical surfaces applications. Neuroimage 318:121370. doi: 10.1016/j.neuroimage.2025.121370 PMC12456340 40669796

[B19] Cong S. Wang H. Zhou Y. Wang Z. Yao X. Yang C. (2024). Comprehensive review of transformer-based models in neuroscience, neurology, and psychiatry. Brain-X 2:e57. doi: 10.1002/brx2.57

[B20] Cui H. Dai W. Zhu Y. Kan X. Gu A. A. C. Lukemire J. . (2023). BrainGB: A benchmark for brain network analysis with graph neural networks. IEEE Trans. Med. Imaging 42, 493–506. doi: 10.1109/TMI.2022.3218745 PMC10079627 36318557

[B21] Dadi K. Rahim M. Abraham A. Chyzhyk D. Milham M. Thirion B. . (2019). Benchmarking functional connectome-based predictive models for resting-state fMRI. Neuroimage 192, 115–134. doi: 10.1016/j.neuroimage.2019.02.062 30836146

[B22] Dai P. Zhou Y. Shi Y. Lu D. Chen Z. Zou B. Liu K. Liao S. REST-meta-MD D. Consortium (2024). Classification of MDD using a transformer classifier with large-scale multisite resting-state fMRI data. Hum. Brain Mapp. 45:e26542. doi: 10.1002/hbm.26542 PMC10789197 38088473

[B23] de Alteriis G. Sherwood O. Ciaramella A. Leech R. Cabral J. Turkheimer F. E. . (2025). DySCo: a general framework for dynamic functional connectivity. PLoS Comput. Biol. 21:e1012795. doi: 10.1371/journal.pcbi.1012795 PMC11902199 40053563

[B24] Deco G. Vidaurre D. Kringelbach M. L. (2021). Revisiting the global workspace orchestrating the hierarchical organization of the human brain. Nat. Human Behav. 5, 497–511. doi: 10.1038/s41562-020-01003-6 PMC8060164 33398141

[B25] Ding J. Dan T. Wei Z. Laurienti P. J. Wu G. (2026). Machine learning on dynamic functional connectivity: Promise, pitfalls, and interpretations. Inf. Sci. 740:123184. doi: 10.1016/j.ins.2026.123184 PMC12927655 41737630

[B26] Dong W. Li Y. Zeng W. Chen L. Yan H. Siok W. T. . (2025). STARFormer: A novel spatio-temporal aggregation reorganization transformer of FMRI for brain disorder diagnosis. Neural Networks 192:107927. doi: 10.1016/j.neunet.2025.107927 40782662

[B27] Dong Z. Li R. Chong J. Dehestani N. Teng Y. Lin Y. . (2025). “Brain harmony: A multimodal foundation model unifying morphology and function into 1D tokens,” in Advances in Neural Information Processing Systems, volume 38, Main Conference, eds. Belgrave D. Zhang C. Lin H. Pascanu R. Koniusz P. Ghassemi M. Chen N. (Red Hook, NY: Curran Associates, Inc.), p. 122100–122127.

[B28] Dosovitskiy A. Beyer L. Kolesnikov A. Weissenborn D. Zhai X. Unterthiner T. . (2021). “An image is worth 16 × 16 words: transformers for image recognition at scale,” in International Conference on Learning Representations. Available online at: https://openreview.net/forum?id=YicbFdNTTy

[B29] Esteban F. J. Vargas E. Langa J. A. Soler-Toscano F. (2026). Synchronization, information, and brain dynamics in consciousness research. Appl. Sci. 16:1056. doi: 10.3390/app16021056

[B30] Fan Z. Su J. Xu J. Zeng L.-L. Hu D. (2024). “A multibranch cross-attention transformer model for psychiatric disorders diagnosis using multi-modal MRI data,” in 2024 8th Asian Conference on Artificial Intelligence Technology (ACAIT) (Piscataway, NJ: IEEE), 126–132.

[B31] Farahani F. V. Fiok K. Lahijanian B. Karwowski W. Douglas P. K. (2022). Explainable AI: A review of applications to neuroimaging data. Front. Neurosci. 16:906290. doi: 10.3389/fnins.2022.906290 PMC9793854 36583102

[B32] Farina M. Ahmad U. Taha A. Younes H. Mesbah Y. Yu X. . (2024). Sparsity in transformers: a systematic literature review. Neurocomputing 582:127468. doi: 10.1016/j.neucom.2024.127468

[B33] Faskowitz J. Betzel R. F. Sporns O. (2022). Edges in brain networks: Contributions to models of structure and function. Netw. Neurosci. 6, 1–28. doi: 10.1162/netn_a_00204 PMC8942607 35350585

[B34] Ferrante M. Iervese S. Astolfi L. Toschi N. (2025). “Self-supervised transformer-based foundation model for functional magnetic resonance imaging,” in 2025 47th Annual International Conference of the IEEE Engineering in Medicine and Biology Society (EMBC) (Copenhagen: IEEE), 1–6. 10.1109/EMBC58623.2025.11254125 41336327

[B35] Gadgil S. Zhao Q. Pfefferbaum A. Sullivan E. V. Adeli E. Pohl K. M. (2020). “Spatio-temporal graph convolution for resting-state fMRI analysis,” in Medical Image Computing and Computer Assisted Intervention-MICCAI 2020, eds. Martel A. L. Abolmaesumi P. Stoyanov D. Mateus D. Zuluaga M. A. Zhou S. K. . (Cham: Springer International Publishing), 528–538. 10.1007/978-3-030-59728-3_52 PMC7700758 33257918

[B36] Gao B. Yu A. Qiao C. Calhoun V. D. Stephen J. M. Wilson T. W. . (2025). An explainable unified framework of spatio-temporal coupling learning with application to dynamic brain functional connectivity analysis. IEEE Trans. Med. Imaging 44, 941–951. doi: 10.1109/TMI.2024.3467384 PMC11977455 39320999

[B37] Gao J. Ge B. Qiang N. Zhao S. (2026). 3D masked autoencoder with spatiotemporal transformer for modeling of 4D fMRI data. Med. Image Anal. 107:103861. doi: 10.1016/j.media.2025.103861 41197226

[B38] Ghayem F. Meudec R. Dockas J. Thirion B. Wassermann D. (2026). NeuroConText: contrastive learning for neuroscience meta-analysis with rich text representation. Imaging Neurosci. 4:IMAG.a.1162. doi: 10.1162/IMAG.a.1162 PMC12994001 41852941

[B39] Ghayem F. Yang H. Kantar F. Kim S.-J. Calhoun V. D. Adali T. (2023). “New interpretable patterns and discriminative features from brain functional network connectivity using dictionary learning,” in ICASSP 2023 - *2023 IEEE International Conference on Acoustics, Speech and Signal Processing (ICASSP)* (Rhodes Island: IEEE), 1–5.

[B40] Gordon E. M. Chauvin R. J. Van A. N. Rajesh A. Nielsen A. Newbold D. J. . (2023). A somato-cognitive action network alternates with effector regions in motor cortex. Nature 617, 351–359. doi: 10.1038/s41586-023-05964-2 PMC10172144 37076628

[B41] Han I. Lee C. Lee J. Ye J. C. (2024). Mindformer: Semantic alignment of multi-subject fmri for brain decoding. arXiv [preprint] arXiv:2405.17720. doi: 10.48550/arXiv.2405.17720

[B42] Han K. Wang Y. Chen H. Chen X. Guo J. Liu Z. . (2023). A survey on vision transformer. IEEE Trans. Pattern Anal. Mach. Intell. 45, 87–110. doi: 10.1109/TPAMI.2022.3152247 35180075

[B43] Han X. Xue R. Feng J. Feng Y. Du S. Shi J. . (2025). Hypergraph foundation model for brain disease diagnosis. IEEE Trans. Neural Netw. Learn. Syst. 36, 17702–17716. doi: 10.1109/TNNLS.2025.3554755 40244841

[B44] Hatamizadeh A. Tang Y. Nath V. Yang D. Myronenko A. Landman B. . (2022). “UNETR: Transformers for 3D medical image segmentation,” in Proceedings of the IEEE/CVF Winter Conference on Applications of Computer Vision (Piscataway, NJ: IEEE), 574–584.

[B45] He K. Gan C. Li Z. Rekik I. Yin Z. Ji W. . (2023). Transformers in medical image analysis. Intellig. Med. 3, 59–78. doi: 10.1016/j.imed.2022.07.002

[B46] He P. Shi Z. Cui Y. Wang R. Wu D. (2024). A spatiotemporal graph transformer approach for Alzheimer's disease diagnosis with rs-fMRI. Comput. Biol. Med. 178:108762. doi: 10.1016/j.compbiomed.2024.108762 38908359

[B47] He Z. Li W. Liu Y. Liu X. Han J. Zhang T. . (2025). FM-APP: Foundation model for any phenotype prediction via fMRI to sMRI knowledge transfer. IEEE Trans. Med. Imaging 44, 4010–4022. doi: 10.1109/TMI.2024.3506734 40030291

[B48] Hu J. Huang Y. Wang N. Dong S. (2024). BrainNPT: Pre-training transformer networks for brain network classification. IEEE Trans. Neural Syst. Rehabilitat. Eng. 32, 2727–2736. doi: 10.1109/TNSRE.2024.3434343 39074019

[B49] Huang H. Zhu Z. Ge Y. Deng R. Chen Z.-M. (2026). Sub-connection learning for fMRI-based brain functional network. IEEE Trans. Neural Syst. Rehabilitat. Eng. 34, 834–844. doi: 10.1109/TNSRE.2026.3658628 41605142

[B50] Iraji A. Deramus T. P. Lewis N. Yaesoubi M. Stephen J. M. Erhardt E. . (2019). The spatial chronnectome reveals a dynamic interplay between functional segregation and integration. Hum. Brain Mapp. 40, 3058–3077. doi: 10.1002/hbm.24580 PMC6548674 30884018

[B51] Islam S. Khanra P. Nakuci J. Muldoon S. F. Watanabe T. Masuda N. (2024). State-transition dynamics of resting-state functional magnetic resonance imaging data: model comparison and test-to-retest analysis. BMC Neurosci. 25:14. doi: 10.1186/s12868-024-00854-3 PMC10913599 38438838

[B52] Izadi M. Safayani M. (2025). ASDFormer: a transformer with mixtures of pooling-classifier experts for robust autism diagnosis and biomarker discovery. arXiv [preprint] arXiv:2508.14005. doi: 10.48550/arXiv.2508.14005

[B53] Jain P. Chakraborty A. Hafiz R. Sao A. K. Biswal B. (2023). Enhancing the network specific individual characteristics in rs-fMRI functional connectivity by dictionary learning. Hum. Brain Mapp. 44, 3410–3432. doi: 10.1002/hbm.26289 PMC10171559 37070786

[B54] Jain S. Wallace B. C. (2019). “Attention is not explanation,” in Proceedings of the 2019 Conference of the North American Chapter of the Association for Computational Linguistics: Human Language Technologies, Volume 1 (Long and Short Papers), eds. Burstein J. Doran C. Solorio T. (Minneapolis, Minnesota: Association for Computational Linguistics), 3543–3556.

[B55] Jazi D. J. Hajiesmaeili M. (2025). Transformers for multimodal brain state decoding: Integrating functional magnetic resonance imaging data and medical metadata. arXiv [preprint] arXiv:2512.08462.

[B56] Jeyabose A. Boerwinkle V. L. Robinson B. Leggio O. Kazemi M. H. (2025). Interpretable transformer models for rs-fMRI epilepsy classification and biomarker discovery. medRxiv [preprint]. doi: 10.1101/2025.09.02.25334737

[B57] Jung S. Lee D. Kim W. H. (2025). “MindLink: Subject-agnostic Cross-Subject Brain Decoding Framework,” in Proceedings of Medical Image Computing and Computer Assisted Intervention-MICCAI 2025, volume LNCS 15971 (Cham: Springer Nature Switzerland), 469–479.

[B58] Kan X. Cui H. Lukemire J. Guo Y. Yang C. (2022a). “FBNETGEN: Task-aware GNN-based fMRI analysis via functional brain network generation,” in Proceedings of The 5th International Conference on Medical Imaging with Deep Learning (New York: PMLR), 618–637. PMC10296778 37377881

[B59] Kan X. Dai W. Cui H. Zhang Z. Guo Y. Yang C. (2022b). Brain network transformer. Adv. Neural Inf. Process. Syst. 35, 25586–25599. doi: 10.52202/068431-1855

[B60] Kannan A. Lindquist M. A. Caffo B. (2025). “BrainMT: a hybrid Mamba-transformer architecture for modeling long-range dependencies in functional MRI data,” in Proceedings of Medical Image Computing and Computer Assisted Intervention-MICCAI 2025, volume LNCS 15971 (Cham: Springer Nature Switzerland), 150–160.

[B61] Kawahara J. Brown C. J. Miller S. P. Booth B. G. Chau V. Grunau R. E. . (2017). BrainNetCNN: Convolutional neural networks for brain networks; towards predicting neurodevelopment. Neuroimage 146, 1038–1049. doi: 10.1016/j.neuroimage.2016.09.046 27693612

[B62] Kazemivash B. Suresh P. Ye D. H. Iraji A. Liu J. Plis S. . (2025). St-DenseViT: a weakly supervised spatiotemporal vision transformer for dense prediction of dynamic brain networks. Hum. Brain Mapp. 46:e70364. doi: 10.1002/hbm.70364 PMC12476115 41014302

[B63] Khajehnejad M. Habibollahi F. Stoliker D. Razi A. (2025). Brainsymphony: a parameter-efficient multimodal foundation model for brain dynamics with limited data. arXiv [preprint] arXiv:2506.18314. doi: 10.48550/arXiv.2506.18314

[B64] Kim B.-H. Ye J. C. Kim J.-J. (2021). Learning dynamic graph representation of brain connectome with spatio-temporal attention. Adv. Neural Inf. Process. Syst. 34, 4314–4327.

[B65] Kim P. Kwon J. Joo S. Bae S. Lee D. Jung Y. . (2023). “SwiFT: Swin 4D fMRI transformer,” in Advances in Neural Information Processing Systems, volume 36, eds. Oh A. Naumann T. Globerson A. Saenko K. Hardt M. Levine S. (Red Hook, NY:Curran Associates, Inc.), 42015–42037.

[B66] Kong Y. Zhang X. Wang W. Zhou Y. Li Y. Yuan Y. (2025). Multi-scale spatial-temporal attention networks for functional connectome classification. IEEE Trans. Med. Imaging 44, 475–488. doi: 10.1109/TMI.2024.3448214 39172603

[B67] Kwon J. Seo J. Wang H. Moon T. Yoo S. Cha J. (2025). Predicting task-related brain activity from resting-state brain dynamics with fMRI transformer. Imaging Neurosci. 3:00440. doi: 10.1162/imag_a_00440 PMC12320000 40800966

[B68] Kwong K. K. Belliveau J. W. Chesler D. A. Goldberg I. E. Weisskoff R. M. Poncelet B. P. . (1992). Dynamic magnetic resonance imaging of human brain activity during primary sensory stimulation. Proc. Natl. Acad. Sci. USA. 89, 5675–5679. doi: 10.1073/pnas.89.12.5675 PMC49355 1608978

[B69] Lane C. Tripathy M. Murali L. K. Grandhi R. S. Yang S. S. Z. Gijsen S. . (2026). “Scaling vision transformers for functional MRI with flat maps,” in ICML (PMLR).

[B70] Le C. Zhao Y. Emami N. Yadav K. Liu X. C. Chen X. Wang Y. (2025). “Voxelformer: Parameter-efficient multi-subject visual decoding from fMRI,” in 2025 12th International IEEE/EMBS Conference on Neural Engineering (NER), 971–975. doi: 10.1109/NER61569.2025.11589005

[B71] Lee J. Kang E. Heo D.-W. Suk H.-I. (2024). Site-invariant meta-modulation learning for multisite autism spectrum disorders diagnosis. IEEE Trans. Neural Netw. Learn. Syst. 35, 18062–18075. doi: 10.1109/TNNLS.2023.3311195 37708014

[B72] Leng Y. Anwar S. M. Rekik I. He S. Lee E.-J. (2025). “Self-supervised graph transformer with contrastive learning for brain connectivity analysis towards improving autism detection,” in 2025 IEEE 22nd International Symposium on Biomedical Imaging (ISBI) (Houston, TX: IEEE), 1–5.

[B73] Li X. Zhou Y. Dvornek N. Zhang M. Gao S. Zhuang J. . (2021). BrainGNN: Interpretable brain graph neural network for fMRI analysis. Med. Image Anal. 74:102233. doi: 10.1016/j.media.2021.102233 PMC9916535 34655865

[B74] Li Y. Ma X. Sunderraman R. Ji S. Kundu S. (2023). Accounting for temporal variability in functional magnetic resonance imaging improves prediction of intelligence. Hum. Brain Mapp. 44, 4772–4791. doi: 10.1002/hbm.26415 PMC10400788 37466292

[B75] Li Z. Wang L. (2026). A dual-stream transformer with self-supervised contrastive training for fMRI-based autism spectrum disorder classification. Brain Sci. 16:277. doi: 10.3390/brainsci16030277 PMC13024321 41892621

[B76] Lin T. Wang Y. Liu X. Qiu X. (2022). A survey of transformers. AI Open 3, 111–132. doi: 10.1016/j.aiopen.2022.10.001

[B77] Lindquist M. A. Geuter S. Wager T. D. Caffo B. S. (2019). Modular preprocessing pipelines can reintroduce artifacts into fmri data. Hum. Brain Mapp. 40, 2358–2376. doi: 10.1002/hbm.24528 PMC6865661 30666750

[B78] Liu P. Dong G. Guo D. Li K. Li F. Yang X. . (2025). A survey on fMRI-based brain decoding for reconstructing multimodal stimuli. arXiv [preprint] arXiv:2503.15978. doi: 10.48550/arXiv.2503.15978

[B79] Lynn C. W. Bassett D. S. (2019). The physics of brain network structure, function and control. Nat. Rev. Phys. 1, 318–332. doi: 10.1038/s42254-019-0040-8

[B80] Mahmood U. Fu Z. Calhoun V. Plis S. (2023). “Glacier: Glass-box transformer for interpretable dynamic neuroimaging,” in ICASSP 2023 - *2023 IEEE International Conference on Acoustics, Speech and Signal Processing (ICASSP)* (Rhodes Island: IEEE), 1–5. 10.1109/icassp49357.2023.10097126 PMC10231935 37266485

[B81] Malkiel I. Rosenman G. Wolf L. Hendler T. (2022). “Self-supervised transformers for fMRI representation,” in Proceedings of The 5th International Conference on Medical Imaging with Deep Learning (New York: PMLR), 895–913.

[B82] Manzari O. N. Ahmadabadi H. Kashiani H. Shokouhi S. B. Ayatollahi A. (2023). MedViT: a robust vision transformer for generalized medical image classification. Comput. Biol. Med. 157:106791. doi: 10.1016/j.compbiomed.2023.106791 36958234

[B83] Marek S. Tervo-Clemmens B. Calabro F. J. Montez D. F. Kay B. P. Hatoum A. S. . (2022). Reproducible brain-wide association studies require thousands of individuals. Nature 603, 654–660. doi: 10.1038/s41586-022-04492-9 PMC8991999 35296861

[B84] Medvedeva T. Knyazeva I. Masharipov R. Korotkov A. Cherednichenko D. Kireev M. (2026). Benchmarking resting state fMRI connectivity pipelines for classification: Robust accuracy despite processing variability in cross-site eye state prediction. Brain Inform. 13:18. doi: 10.1186/s40708-026-00305-1 PMC13197499 42084762

[B85] Menon V. (2023). 20 years of the default mode network: a review and synthesis. Neuron 111, 2469–2487. doi: 10.1016/j.neuron.2023.04.023 PMC10524518 37167968

[B86] Mohammadi H. Karwowski W. (2025). Graph neural networks in brain connectivity studies: methods, challenges, and future directions. Brain Sci. 15:17. doi: 10.3390/brainsci15010017 PMC11763835 39851385

[B87] Munroe L. Da Silva M. Heidari F. Grigorescu I. Dahan S. Robinson E. C. . (2024). Applications of interpretable deep learning in neuroimaging: a comprehensive review. Imag. Neurosci. 2:imag-2-00214. doi: 10.1162/imag_a_00214 PMC12272211 40800540

[B88] Ngo G. H. Nguyen M. Chen N. F. Sabuncu M. R. (2022). A transformer-based neural language model that synthesizes brain activation maps from free-form text queries. Med. Image Anal. 81:102540. doi: 10.1016/j.media.2022.102540 35914394

[B89] Nguyen X.-B. Li X. Sinha P. Khan S. U. Luu K. (2025). Brainformer: Mimic human visual brain functions to machine vision models via fMRI. Neurocomputing 620:129213. doi: 10.1016/j.neucom.2024.129213

[B90] Nie J. Han K. Zhang T. You C. Van Rooij S. Stevens J. . (2025). “Brain network state transformer: Leveraging state functional connectivity for enhanced brain network analysis,” in 2025 47th Annual International Conference of the IEEE Engineering in Medicine and Biology Society (EMBC) (Copenhagen: IEEE), 1–7. 10.1109/EMBC58623.2025.11254044 41337146

[B91] Ortega Caro J. de Oliveira Fonseca A. H. Rizvi S. Rosati M. Averill C. Cross J. . (2024). “BrainLM: A foundation model for brain activity recordings,” in International Conference on Learning Representations, volume 2024, eds. Kim B. Yue Y. Chaudhuri S. Fragkiadaki K. Khan M. Sun Y., 565–576.

[B92] Pang J. C. Aquino K. M. Oldehinkel M. Robinson P. A. Fulcher B. D. Breakspear M. . (2023). Geometric constraints on human brain function. Nature 618, 566–574. doi: 10.1038/s41586-023-06098-1 PMC10266981 37258669

[B93] Preti M. G. Bolton T. A. Van De Ville D. (2017). The dynamic functional connectome: state-of-the-art and perspectives. Neuroimage 160, 41–54. doi: 10.1016/j.neuroimage.2016.12.061 28034766

[B94] Rahman M. M. Calhoun V. Plis S. (2026). Deep learning interpretability in neuroimaging: a comprehensive survey and methodological recommendations. Imag. Neurosci. 4:IMAG-a. doi: 10.1162/IMAG.a.1129 PMC12977092 41821831

[B95] Raza W. H. Bader Shah A. Wen Y. Shen Y. Martinez Lemus J. D. Caryn Schiess M. . (2025). “NeuroMoE: a transformer-based mixture-of-experts framework for multi-modal neurological disorder classification,” in 2025 47th Annual International Conference of the IEEE Engineering in Medicine and Biology Society (EMBC) (Copenhagen: IEEE), 1–7. 10.1109/EMBC58623.2025.11254303 41336692

[B96] Ren J. An N. Lin C. Zhang Y. Sun Z. Zhang W. . (2025). DeepPrep: An accelerated, scalable and robust pipeline for neuroimaging preprocessing empowered by deep learning. Nat. Methods 22, 473–476. doi: 10.1038/s41592-025-02599-1 PMC11903312 39915693

[B97] Ren Y. Ding Z. Yang R. Wang K. Sun K. Li X. . (2024). “Modelling spatio-temporal features of task FMRI data via spatio-temporal fusion-transformer,” in 2024 IEEE International Symposium on Biomedical Imaging (ISBI) (Athens: IEEE), 1–4.

[B98] Sadil P. Lindquist M. A. (2026). From maps to models: a survey on the reliability of small studies of task-based fMRI. Imag. Neurosci. 4:IMAG.a.1076. doi: 10.1162/IMAG.a.1076 PMC12779752 41523208

[B99] Sarraf S. Sarraf A. DeSouza D. D. Anderson J. A. E. Kabia M. Initiative T. A. D. N. (2023). OViTAD: Optimized vision transformer to predict various stages of Alzheimer's disease using resting-state fMRI and structural MRI data. Brain Sci. 13:260. doi: 10.3390/brainsci13020260 PMC9954686 36831803

[B100] Scarciglia A. Catrambone V. Bianco M. Bonanno C. Toschi N. Valenza G. (2024). Stochastic brain dynamics exhibits differential regional distribution and maturation-related changes. Neuroimage 290:120562. doi: 10.1016/j.neuroimage.2024.120562 38484917

[B101] Schaefer A. Kong R. Gordon E. M. Laumann T. O. Zuo X.-N. Holmes A. J. . (2018). Local-global parcellation of the human cerebral cortex from intrinsic functional connectivity MRI. Cereb. Cortex 28, 3095–3114. doi: 10.1093/cercor/bhx179 PMC6095216 28981612

[B102] Seguin C. Sporns O. Zalesky A. (2023). Brain network communication: Concepts, models and applications. Nat. Rev. Neurosci. 24, 557–574. doi: 10.1038/s41583-023-00718-5 37438433

[B103] Seo J. Park D. K. Yoo S. Cha J. (2025). “Scalable diffusion transformer for conditional 4d fMRI synthesis,” in NeurIPS 2025 Workshop on Foundation Models for the Brain and Body.

[B104] Shamshad F. Khan S. Zamir S. W. Khan M. H. Hayat M. Khan F. S. . (2023). Transformers in medical imaging: a survey. Med. Image Anal. 88:102802. doi: 10.1016/j.media.2023.102802 37315483

[B105] Shehzad A. Zhang D. Yu S. Abid S. Xia F. (2025). Dynamic graph transformer for brain disorder diagnosis. IEEE J. Biomed. Health Inform. 29, 4388–4400. doi: 10.1109/JBHI.2025.3538040 40031384

[B106] Shen G. Zhao D. He X. Feng L. Dong Y. Wang J. . (2024). Neuro-vision to language: Enhancing brain recording-based visual reconstruction and language interaction. Adv. Neural Inf. Process. Syst. 37, 98083–98110. doi: 10.52202/079017-3113

[B107] Sheng J. Wang L. Cheng H. Zhang Q. Zhou R. Shi Y. (2021). Strategies for multivariate analyses of imaging genetics study in Alzheimer's disease. Neurosci. Lett. 762:136147. doi: 10.1016/j.neulet.2021.136147 34332030

[B108] Shi C. Wang Y. Wu Y. Chen S. Hu R. Zhang M. . (2023). Self-supervised pretraining improves the performance of classification of task functional magnetic resonance imaging. Front. Neurosci. 17–2023. doi: 10.3389/fnins.2023.1199312 PMC10330812 37434766

[B109] Shi L. Shi L. Cui Z. Lin C. Zhang R. Zhang J. . (2025). Transformer-based structural connectivity networks for ADHD-related connectivity alterations. Commun. Med. 5:296. doi: 10.1038/s43856-025-01015-1 PMC12271470 40676171

[B110] Song X. Shu K. Yang P. Zhao C. Zhou F. Frangi A. F. . (2025). Knowledge-aware multisite adaptive graph transformer for brain disorder diagnosis. IEEE Trans. Med. Imaging 44, 2370–2383. doi: 10.1109/TMI.2024.3453419 39222450

[B111] Sun Y. Cabezas M. Lee J. Wang C. Zhang W. Calamante F. . (2025a). “Predicting human brain states with transformer,” in Medical Image Computing and Computer Assisted Intervention-MICCAI 2024 Workshops, eds. Schroder A. Li X. Syeda-Mahmood T. Oxtoby N. P. Young A. Hering A. . (Cham: Springer Nature Switzerland), 136–146.

[B112] Sun Y. Chahine D. Wen Q. Liu T. Li X. Yuan Y. . (2025b). Voxel-level brain states prediction using swin transformer. IEEE J. Biomed. Health Inform. 29, 8719–8726. doi: 10.1109/JBHI.2025.3613793 41359725

[B113] Tang J. Du M. Vo V. Lal V. Huth A. (2023). Brain encoding models based on multimodal transformers can transfer across language and vision. Adv. Neural Inf. Process. Syst. 36, 29654–29666. doi: 10.52202/075280-1290 PMC11250991 39015152

[B114] Tang J. Zhu T. Zhou W. Zhao W. (2026). Graph neural networks for fMRI functional brain networks: a survey. Neural Netw. 194:108137. doi: 10.1016/j.neunet.2025.108137 41066997

[B115] Thapaliya B. Miller R. Chen J. Wang Y. P. Akbas E. Sapkota R. . (2025). DSAM: A deep learning framework for analyzing temporal and spatial dynamics in brain networks. Med. Image Anal. 101:103462. doi: 10.1016/j.media.2025.103462 PMC13060574 39892220

[B116] Thomas Yeo B. T. Krienen F. M. Sepulcre J. Sabuncu M. R. Lashkari D. Hollinshead M. . (2011). The organization of the human cerebral cortex estimated by intrinsic functional connectivity. J. Neurophysiol. 106, 1125–1165. doi: 10.1152/jn.00338.2011 PMC3174820 21653723

[B117] Tian X. Ma H. Guan Y. Xu L. Liu J. Tian L. (2025). Transformer3: A pure transformer framework for fMRI-based representations of human brain function. IEEE J. Biomed. Health Inform. 29, 468–481. doi: 10.1109/JBHI.2024.3471186 39365723

[B118] Vaswani A. Shazeer N. Parmar N. Uszkoreit J. Jones L. Gomez A. N. . (2017). “Attention is all you need,” in Advances in Neural Information Processing Systems, volume 30 (Red Hook, NY:Curran Associates, Inc.).

[B119] Vidaurre D. (2024). Dynamic functional connectivity: why the controversy? Imag. Neurosci. 2:imag-2-00363. doi: 10.1162/imag_a_00363 PMC12315744 40801399

[B120] Wang B. (2025). ViT transfer learning for fMRI (VTFF): a highway to achieve superior performance for multi-classification of cognitive decline. Biomed. Signal Process. Control 104:107557. doi: 10.1016/j.bspc.2025.107557

[B121] Wang C. Jiang Y. Peng Z. Li C. Bang C.-B. Zhao L. . (2026). Towards a general-purpose foundation model for functional MRI analysis. Nat. Biomed. Eng. doi: 10.1038/s41551-026-01666-y 42026120

[B122] Wang J. Wu L. Kuang H. (2024). “Local-global spatio-temporal encoding with adaptive brain region signal segmentation for mild cognitive impairment detection,” in 2024 IEEE International Conference on Bioinformatics and Biomedicine (BIBM) (Lisbon: IEEE), 2639–2644.

[B123] Wang L. Li K. Hu X. P. (2021). Graph convolutional network for fMRI analysis based on connectivity neighborhood. Netw. Neurosci. 5, 83–95. doi: 10.1162/netn_a_00171 PMC7935029 33688607

[B124] Wang M. Guo J. Guo X. (2026a). A multi-atlas dynamic connectivity transformer fused with 4D spatiotemporal modeling for autism spectrum disorder recognition. Brain Sci. 16:378. doi: 10.3390/brainsci16040378 PMC13114882 42041790

[B125] Wang M. Lian C. Yao D. Zhang D. Liu M. Shen D. (2020). Spatial-temporal dependency modeling and network hub detection for functional MRI analysis via convolutional-recurrent network. IEEE Trans. Biomed. Eng. 67, 2241–2252. doi: 10.1109/TBME.2019.2957921 PMC7439279 31825859

[B126] Wang M. Xia J. Ye W. Liu E. Peng K. Feng J. . (2026b). SLIM-brain: a data- and training-efficient foundation model for fMRI data analysis. arXiv [preprint] arXiv:2512.21881. doi: 10.48550/arXiv.2512.21881

[B127] Wang M. Ye W. Xia J. Zhang J. Pan X. Xu M. . (2026c). “Omni-fMRI: a universal atlas-free fMRI foundation model,” in Forty-Third International Conference on Machine Learning (PMLR).

[B128] Wang M. Zhu L. Li X. Pan Y. Li L. (2023). Dynamic functional connectivity analysis with temporal convolutional network for attention deficit/hyperactivity disorder identification. Front. Neurosci. 17:1322967. doi: 10.3389/fnins.2023.1322967 PMC10750397 38148943

[B129] Wang W. Chen C. Ding M. Yu H. Zha S. Li J. (2021). “TransBTS: Multimodal brain tumor segmentation using transformer,” in Medical Image Computing and Computer Assisted Intervention-MICCAI 2021, eds. de Bruijne M. Cattin P. C. Cotin S. Padoy N. Speidel S. Zheng Y. Essert C. (Cham: Springer International Publishing), 109–119.

[B130] Wang Y. Wang S. Cai W. Ford G. Cui Y. Zhang Y. . (2026). Representation, alignment, and generation: A comprehensive survey of foundation models for non-invasive brain decoding. bioRxiv [preprint]. doi: 10.64898/2025.11.30.691403

[B131] Watters H. Fazili A. Daley L. Belden A. LaGrow T. J. Bolt T. . (2024). Creative tempo: spatiotemporal dynamics of the default mode network in improvisational musicians. *bioRxiv* [preprint]. doi: 10.1101/2024.04.07.588391

[B132] Wei X. Zhao K. Jiao Y. He L. Zhang Y. (2026). A brain graph foundation model: Pre-training and prompt-tuning across broad atlases and disorders. arXiv [preprint] arXiv:2506.02044. doi: 10.48550/arXiv.2506.02044

[B133] Wei Z. Dan T. Chen T. Wu G. (2025). “BrainMoE: Cognition joint embedding via mixture-of-expert towards robust brain foundation model,” in Advances in Neural Information Processing Systems, volume 38, Main Conference, eds. Belgrave D. Zhang C. Lin H. Pascanu R. Koniusz P. Ghassemi M. Chen N. (Red Hook, NY:Curran Associates, Inc.), 4541–4563.

[B134] Wei Z. Dan T. Ding J. Wu G. (2024). Neuropath: A neural pathway transformer for joining the dots of human connectomes. Adv. Neural Inf. Process. Syst. 37, 67826–67849. doi: 10.52202/079017-2166

[B135] Wiegreffe S. Pinter Y. (2019). “Attention is not not explanation,” in Proceedings of the 2019 Conference on Empirical Methods in Natural Language Processing and the 9th International Joint Conference on Natural Language Processing (EMNLP-IJCNLP), eds. Inui K. Jiang J. Ng V. Wan X. (Hong Kong, China: Association for Computational Linguistics), 11–20.

[B136] Wu K. Jelfs B. Neville K. Cai A. Fang Q. (2024). fMRI-based static and dynamic functional connectivity analysis for post-stroke motor dysfunction patient: a review. IEEE Access 12, 133067–133085. doi: 10.1109/ACCESS.2024.3445580

[B137] Xia Y. Arab F. Saha U. Sipes B. Gooden G. Chen M. . (2026). MAMBAxBrain: A multi-task neural framework linking brain functional dynamics to individual fingerprints, cognitive and disease states. bioRxiv [preprint]. doi: 10.64898/2026.02.08.704658

[B138] Xue R. Han X. Hu H. Zhang Z. Du S. Gao Y. (2025). “Adaptive embedding for long-range high-order dependencies via time-varying transformer on fMRI,” in Proceedings of Medical Image Computing and Computer Assisted Intervention-MICCAI 2025, volume LNCS 15971 (Cham: Springer Nature Switzerland), 46–55.

[B139] Yu G. Hu X. Aviles-Rivero A. I. Qiu A. Wang S. (2026). Moving beyond functional connectivity: time-series modeling for fMRI-based brain disorder classification. IEEE Trans. Med. Imaging 45, 2764–2779. doi: 10.1109/TMI.2026.3662157 41650432

[B140] Yu M. (2026). Leveraging local and global features: A CNN-transformer hybrid for fMRI-based autism diagnosis. Informatica 50. doi: 10.31449/inf.v50i5.12777

[B141] Yu X. Zhang L. Zhao L. Lyu Y. Liu T. Zhu D. (2022). Disentangling spatial-temporal functional brain networks via twin-transformers. arXiv [preprint] arXiv:2204.09225. doi: 10.48550/arXiv.2204.09225

[B142] Zhang H. Song C. Zhao X. Wang F. Qiu Y. Li H. . (2024). STDCformer: Spatial-temporal dual-path cross-attention model for fMRI-based autism spectrum disorder identification. Heliyon 10:e34245. doi: 10.1016/j.heliyon.2024.e34245 PMC11734066 39816341

[B143] Zhang J. Lyu Y. Yu X. Zhang L. Cao C. Chen T. . (2025a). “Classiffication of mild cognitive impairment based on dynamic functional connectivity using spatio-temporal transformer,” in 2025 IEEE 22nd International Symposium on Biomedical Imaging (ISBI) (Houston, TX: IEEE), 1–5. 10.1109/isbi60581.2025.10980922 PMC12715849 41425905

[B144] Zhang J. Wu X. Tang X. Zhou L. Wang L. Wu W. . (2025b). Asynchronous functional brain network construction with spatiotemporal transformer for MCI classification. IEEE Trans. Med. Imaging 44, 1168–1180. doi: 10.1109/TMI.2024.3486086 39446548

[B145] Zhang S. Jiang Z. Shen X. Yu Z. Chen X. Zhang X.-Y. . (2025c). “Graph disentanglement learning for fMRI analysis: decoupling disease, covariates, and individual variability,” in Proceedings of Medical Image Computing and Computer Assisted Intervention-MICCAI 2025, volume LNCS 15971 (Cham: Springer Nature Switzerland), 352–361.

[B146] Zhao L. (2025). Advances in functional magnetic resonance imaging-based brain function mapping: a deep learning perspective. Psychoradiology 5:kkaf007. doi: 10.1093/psyrad/kkaf007 PMC12093097 40401160

[B147] Zheng C. El-Sappagh S. Abuhmed T. (2026). 4DfCF: 4D fMRI CrossFormer vision transformer. IEEE J. Biomed. Health Inform. 30:5114–5127. doi: 10.1109/JBHI.2025.3631655 41231696

[B148] Zhou J. Jie B. Wang Z. Zhang Z. Du T. Bian W. . (2024). Lcgnet: Local sequential feature coupling global representation learning for functional connectivity network analysis with fmri. IEEE Trans. Med. Imaging 43, 4319–4330. doi: 10.1109/TMI.2024.3421360 38949932

[B149] Zhou Y. Duan P. Du Y. Dvornek N. C. (2025). “Self-supervised pre-training tasks for an fMRI time-series transformer in autism detection,” in Machine Learning in Clinical Neuroimaging, eds. Bathula D. R. Benet Nirmala A. Dvornek N. C. Govindarajan S. T. Habes M. (Cham: Springer Nature Switzerland), 145–154. 10.1007/978-3-031-78761-4_14 PMC11951341 40160559

[B150] Zongxin D. Lin L. (2024). “Braintransformer: Subject-wise patch embed transformer for cross-subject brain visual information decoding,” in 2024 21st International Computer Conference on Wavelet Active Media Technology and Information Processing (ICCWAMTIP) (Piscataway, NJ: IEEE), 1–4.

